# A Review of Single-Cell Adhesion Force Kinetics and Applications

**DOI:** 10.3390/cells10030577

**Published:** 2021-03-05

**Authors:** Ashwini Shinde, Kavitha Illath, Pallavi Gupta, Pallavi Shinde, Ki-Taek Lim, Moeto Nagai, Tuhin Subhra Santra

**Affiliations:** 1 Department of Engineering Design, Indian Institute of Technology Madras, Chennai 600036, Tamil Nadu, India; ashwiniss90@gmail.com (A.S.); kavithaillath24@gmail.com (K.I.); pgupta1304@gmail.com (P.G.); shindepallavi25@gmail.com (P.S.); 2 Department of Biosystems Engineering, Kangwon National University, Chuncheon-Si, Gangwon-Do 24341, Korea; ktlim@kangwon.ac.kr; 3 Department of Mechanical Engineering, Toyohashi University of Technology, 1-1 Hibarigaoka, Tempaku-cho, Toyohashi, Aichi 441-8580, Japan; nagai@me.tut.ac.jp

**Keywords:** single-cell adhesion, mechanotransduction, microbial cell adhesion, single-molecule adhesion

## Abstract

Cells exert, sense, and respond to the different physical forces through diverse mechanisms and translating them into biochemical signals. The adhesion of cells is crucial in various developmental functions, such as to maintain tissue morphogenesis and homeostasis and activate critical signaling pathways regulating survival, migration, gene expression, and differentiation. More importantly, any mutations of adhesion receptors can lead to developmental disorders and diseases. Thus, it is essential to understand the regulation of cell adhesion during development and its contribution to various conditions with the help of quantitative methods. The techniques involved in offering different functionalities such as surface imaging to detect forces present at the cell-matrix and deliver quantitative parameters will help characterize the changes for various diseases. Here, we have briefly reviewed single-cell mechanical properties for mechanotransduction studies using standard and recently developed techniques. This is used to functionalize from the measurement of cellular deformability to the quantification of the interaction forces generated by a cell and exerted on its surroundings at single-cell with attachment and detachment events. The adhesive force measurement for single-cell microorganisms and single-molecules is emphasized as well. This focused review should be useful in laying out experiments which would bring the method to a broader range of research in the future.

## 1. Introduction

Within a living organism, cells gather information about their surroundings and process them by chemical, electrical and mechanical signals. Chemical and electrical signals are well understood; however, there is much need to learn about mechanical signaling. Along with the traditional knowledge of chemical and electric signaling as the primary mechanism, now mechanical signaling is also known to play an essential role in a vast range of biological activities. Many cells such as immune cells [1], neurons [2], endothelial cells [3], muscle cells [4] and osteocytes [5] are mechanically sensitive, and thus, they generate force. The process known as mechanotransduction is the force between the cell and its surrounding that transmits mechanical signals which are converted into biochemical signals. Mechanotransduction and mechanical activities play a central role in various cell processes such as cell growth [6], differentiation [7], meiosis and mitosis [8], apoptosis [9] and homeostasis [10]. Thus, the malfunction of mechanical stimuli sensed by the cell can have severe consequences which lead to diseases, such as vascular diseases [11], kidney diseases [12], dystrophy [13] and cancer [14]. Thus, it is imperative to characterize and understand mechanical signaling to the same extent as chemical and electrical signaling.

**Figure 1:**
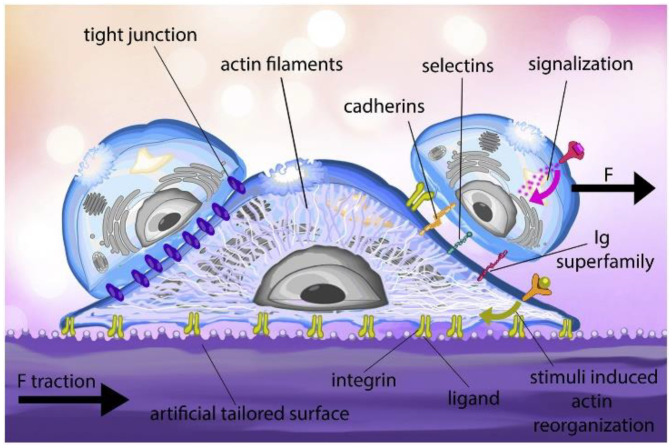
Cell adhesion process: the ligand on the artificially tailored surface binds to the integrin receptors found on the cell membrane. Throughout the adhesion process, the actin filament structure of the cell is reorganized, and a traction force is generated in the substrate. External stimuli also regulate the reorganization of cytoskeletal. After surface adhesion, the cell can interact with other cells through membrane proteins such as cadherins, selectins and the Immunoglobulin (Ig) superfamily. In tissues, cell junctions, a variety of multiprotein complexes (e.g., tight junctions), can form between cells to promote intercellular communication and mechanical stability. (F—Force applied by cell on its surrounding) (Reproduced with permission from [18]).

The study of single-cell adhesion is one of the most important and complicated aspects to understand in life sciences. Different cells adhere to their surrounding surfaces which help them to survive. It also helps in other vital cellular processes such as embryogenesis, cell orientation, morphogenesis, cell motility, immune responses, development, and reorganization. The process involves a multitude of factors present both intrinsic and extrinsic to the cell membrane, such as the cytoskeleton [15], membrane-bound adhesion proteins [16] and glycocalyx elements [17]. The transmembrane proteins, e.g., integrins, form adhesion sites to anchor between the cell and matrix or the other cell’s adhesion molecule. These adhesion molecules are attached to the cytoskeleton, the actin filament through the focal adhesion (FA) complex, which is highly organized with a cluster of molecules (Figure 1).

The passive cell adhesion process is an in vitro process in static medium culture (e.g., culture flasks or petri dishes), where cells undergo morphologic alterations driven by passive deformation and dynamic reorganization of the cytoskeleton. In in vitro conditions, cell adhesion progresses using passive adsorption to the surface, where the initial contact is made by the cell glycocalyx coat, followed by attachment, the spreading of the cell and the formation of focal adhesions. A recent study by Kanyo et al. discovered that the components of the glycocalyx could regulate the speed and magnitude of adhesion [19]. Moreover, an increase and decrease in adhesion can be achieved by using their technique. This is further modulated by the signalization process [20], flow circulation [21] or the cell-extracellular matrix (ECM) under blood flow in in vivo conditions, known to be a dynamic process. The in vivo dynamic cell adhesion is mediated through molecular bonding along with the non-covalent interactions between cell-surface receptors and ligands ECM. The in vivo cell adhesion cascade and signaling events involve two main phases—the docking phase (occurs between the rolling of cells to endothelial cells and to cell arrest) and the locking phase (consists of firm adhesion to the transmigration of the cell) [22]. A wide range of receptors is expressed on the surface of the cells, which helps to bind different ligands with varying affinity. The longer the cell adhesion time to the substrate, the stronger the adhesion strength, which is directly proportional to the number of integrin-ligand pairs, thus increasing the overall contact time. The lateral spacing of ligands, substrate rigidity, ligand tether length, and other factors are responsible for the adhesion strength [23]. The glycocalyx, consisting of glycolipids, proteoglycans, glycoproteins, and polysaccharides, is also involved in the cell adhesion process in addition to integrins [24]. The understanding of cell-surface interaction will help develop any devices or materials for applications in biology and biomedical purposes, as it is necessary to have the medical device in the body compatible with the surrounding tissue. Thus, it will benefit scaffold-based tissue engineering and medical fields.

Over the years, numerous studies have shown the use of different techniques for the analysis of single-cell adhesion force. Approximately 1300 research articles have been published during 2000–2020. The purpose of this focused review is to highlight various commonly used and recent advances in the techniques to analyze single-cell adhesion and understand different fields of study, including biomaterials, the effect of environmental stimuli and biochemical treatments, and adhesion properties of normal and cancer cells. It also addresses the existing limitations of the current techniques and improved aspects such as sensitivity, robustness, reliability, and sample throughput of the new cutting-edge technology. We also showcase the importance of single-molecule adhesion and various tools used for the broader perspective of organ-on-a-chip studies. Despite the pieces of evidence and extraordinary efforts of applying microscopes to detect the pathological alterations of cells, there are still challenges that remain unsolved, such as the need to build more customized setups that would help in high-potential elucidations of mechanisms in biology.

## 2. Single-Cell Mechanical Properties for Mechanotransduction Studies

**Figure 2:**
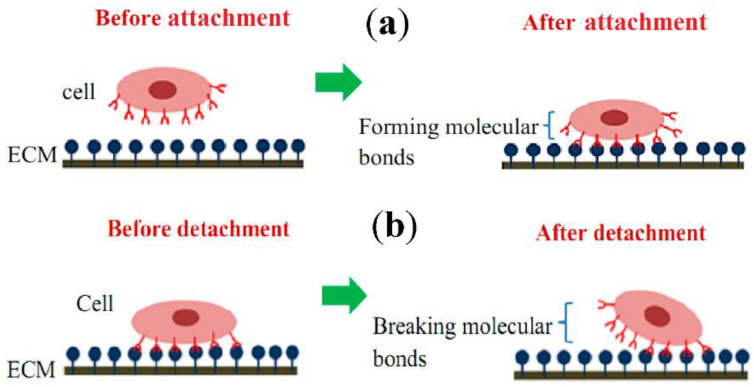
Schematic diagram of single-cell adhesion (**a**) attachment event via the formation of molecular bonds, (**b**) detachment event via breakage of molecular bonds. (ECM—Extracellular matrix) (Reproduced with permission from [25] under Creative Commons Attribution 4.0 International License).

Cell-ECM mechanical interactions influence cell behavior and function. Tremendous interest has been created in developing various methods for measuring cellular mechanical properties in physiology and diseases. Numerous techniques have been designed for cell adhesion studies at the single-cell as well as population level. Here, we have categorized cell adhesion into cell attachment (cell generated forces) and detachment events (ECM mechanical forces), particularly for single-cell studies. Figure 2 shows that single-cell adhesion attachment events are focused on the cell-substrate attachment mechanism via the formation of molecular bonds. In contrast, the detachment events involve the application of force and breakage of molecular bonds to detach the adhered cell from the substrate. Table 1 summarizes the advantages and limitations of techniques used to study single-cell adhesion.

### 2.1. Measurement of Cell Generated Forces

Cell generated forces play an essential role in the development and function of microvascular networks. In many cases, cells use these forces to sense the physical nature of the surrounding to initiate signaling cascades that would influence the cell behavior and phenotype. In cell attachment studies, various covalent and non-covalent molecular bonds are formed between the cell surface receptors and the complementary ligands on the ECM surface (Figure 2a), which help us to observe and study their responses through different behavior and morphological changes. For single-cell study, the experiments were performed to measure and analyze the cell-substrate interaction forces using traction force microscopy (TFM) and the micropatterning technique. It is crucial to study the adhesion behavior of cells towards different treatments and physiological conditions, the determination of biocompatibility of other materials for tissue engineering, cell adhesion kinetics, cancer cell metastasis and drug treatment studies.

#### 2.1.1. Traction Force Microscopy

**Figure 3:**
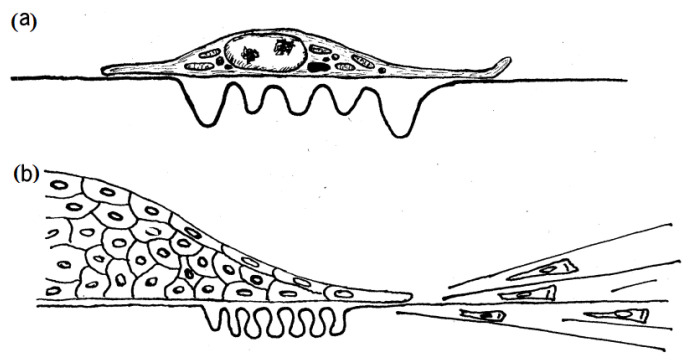
Diagrammatic view of traction force exerted by cell. (**a**) Diagrammatic side view of an individually cultured fibroblast distorting and wrinkling the elastic silicone substratum upon which it has spread and crawled. (**b**) Diagrammatic side view of the margin of an explant whose cells are spreading outward on a silicone rubber substratum. The traction forces exerted by the outgrowing cells compress the rubber sheet beneath the explant and stretch it into long radial wrinkles in the surrounding area. (Reproduced with permission from [26]).

Traction force microscopy (TFM) is the first technique, which was developed to measure the tractions exerted on the soft elastic substrate by the single-cell and tissues. The first implementation using TFM showed that the *single-cell* is able to wrinkle a smooth, thin silicon rubber substrate to which it has adhered Figure 3a,b) [26]. Various improvements were attempted to quantify the tractions for such deformation by fabricating substrates as a thin, flat membrane. TFM at the single-cell level has made visible the tractions that cells exert when they divide, migrate, or interact with their environment. At the tissue level, TFM has been used to coordinate the local tractions generated during collective cell migration, the mechanism of wave propagation in the cell monolayer [27] and cells motility mode in wound healing [28]. Other discoveries by TFM include kenotaxis (cellular traction forces by communal polarization), cell jamming (transition from fluid-like to solid-like collective behavior) and durotaxis (collective migration up an ECM stiffness gradient). Moreover, the TFM has been applied for the tissue explants along with the cultured cells [29].

**Figure 4:**
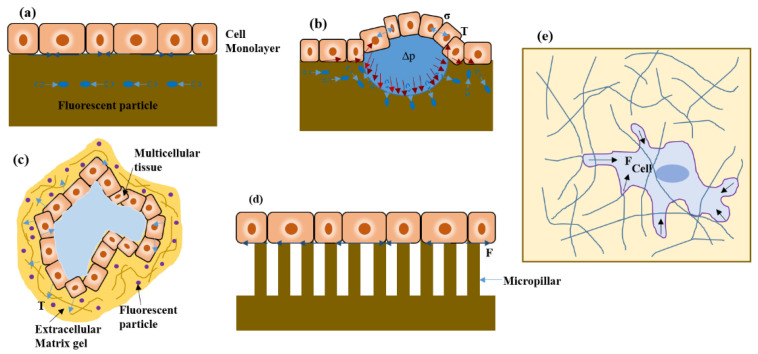
Measurement of cell generated forces. (**a**) Schematic of the principles of 2D traction force microscopy (TFM). Cells or tissue can attach to the surface of a flat elastic gel. Tractions are exerted by the cells on the substrate, and fluorescent particles in or on the substrate are imaged using various microscopy methods, typically, confocal microscopy. The resulting deformation is tracked by comparing the position of the particles with an image of the substrate at rest (that is, after complete removal of adhered cells). Using different computational and analytical approaches, tractions are calculated. (**b**) Schematic of 2.5D TFM. In this technique, cells or tissue are seeded on top of a 2D elastic substrate. The substrate displacements (detected as changes in the position of the blue particles embedded in the substrate, typically by confocal microscopy) are measured in 3D. For simple geometries, such as spherical caps, the internal stresses of the tissue can be recovered with a micro bulge test. (**c**) Schematic of 3D TFM. The 3D displacement field (blue arrows) for tissues grown inside a deformable ECM gel can be measured by detecting changes in the position of matrix-embedded particle tracers using confocal microscopy. (T—The force per unit area acting on any internal or external surface of a material is called the traction vector T) (**d**) Schematic of the micropillar-array technique. Cells are seeded on top of an array of micropillars. The deflection of these pillars is proportional to the locally applied force. (Figure redrawn from [29]). (**e**) In 3D ECM, cellular tractions are distributed throughout the 3D space, and traction forces propagated along ECM fibers cause remodeling of the ECM, altering local mechanical properties. (F—Force exerted by the cell on the environment) (Figure redrawn from [36]).

The technique was reformulated to 2D-TFM (Figure 4a), its current implementation, where the 2D tractions exerted by the cells on the flat substrates with known thickness were measured. The substrates typically used are made of soft polydimethylsiloxane (PDMS) gels and polyacrylamide (PA), which are tunable in stiffness, transparent and able to be coated with ECM proteins. Numerous studies have used 2D-TFM to directly measure the cell displacement generated on the upper surface of the substrate to which they are adhered, along with the fluorescent beads embedded near the gel surface [30]. The displacement is measured upon the cell adhesion attachment event, where the traction force is generated by the cell and quantified by tracking the displacement of the fluorescent beads. This is relative to the reference state, obtained by detaching all the cells from the substrate and thus relaxing it to its non-deformed configuration. These tractions exerted on the substrate are computed using light microscopy by the imaging of known markers that are attached to its surface or embedded in the substrate. Recently, the 2D-TFM technique has eliminated the need for theoretical reference [31]. Razafiarison et al. used the polyacrylic acid (PAA) hydrogel-based TFM method to reveal the relationship of human mesenchymal stem cells (hMSCs) to matrix stiffness with the self-assembly of supramolecule and ECM ligand topology with respect to surface energy on the surface of the biomaterial [32,33]. Such a relationship responsible for stem cell lineage commitment was determined by the TFM. Moreover, cell migration, traction force and focal adhesion dynamics were studied by Onochie et al., after corneal injury, using a three model system to determine the alteration in substrate stiffness by cell migration [34]. Here, cell motility and traction forces were analyzed using fibronectin-coated 8 and 30 kPa polyacrylamide substrates for epithelial sheets culture. The demonstrated experiments resulted in the contribution of change in substrate stiffness affected by traction forces and vinculin dynamics to the delayed healing response in corneal wounds.

Cells induce 3D displacement on the ECM by applying 3D forces, even when attached to the flat surfaces. In such cases, 2.5D-TFM (Figure 4b) measures the 3D displacement of the top surface of the substrate and also relaxes the zero normal traction hypothesis at the substrate surface. Thus, 2.5D-TFM is used to explain the interplay between tangential and normal forces during the migration of single-cells and leukocyte diapedesis through the vascular endothelial monolayer. While the 3D traction force is influenced in the protease-dependent invasion of cancer. The main benefit of 2.5D-TFM is that it has retained its analytical and experimental simplicity from 2D-TFM while qualitatively improving the scope of its measurement by quantifying 3D tractions [29]. While deciphering physiological processes, such as angiogenesis or tumor invasion, it becomes necessary to measure in 3D traction forces exerted by tissue embedded in the ECM. Therefore, this provides a tool to understand in vivo cellular behaviors, such as differentiated function or the maintenance of stem cell function. The 3D-TFM (Figure 4c) uses 3D matrices such as collagen, fibrin, agarose, hyaluronic acid or Matrigel for the cell culture. It is a technique where the displacement of cells is measured using fluorescent beads surrounding the cell, embedded inside the gel matrix [35]—the deflection in pillars within microtissue is the result of the sum of all cell-generated forces [36]. Cells generate contractile forces and impart traction forces normal to the substrate surface in addition to in-plane forces (Figure 4e). Thus, the single-cell contractility in the microtissue is estimated by the total contractility divided by the number of cells present, assuming that the forces are homogeneously distributed throughout the tissue, and most cells are parallelly aligned to the long axis of the tissue in two-pillar uniaxial microtissues [37]. Cell migration through the 3D networks of ECM proteins is possible as compared to the 2D when sufficient traction is generated by the cell to overcome the steric hindrance of the surroundings [25,38]. Cellular contractility has been analyzed using 3D-TFM in a collagen matrix for various cell lines, for example, human foreskin fibroblasts [39], NIH/3T3 fibroblasts [40], cardiomyocytes [41], valvular interstitial cells [42], and lung fibroblasts [43]. Based on the cell type, culture conditions and force measurement method, the force generated per cell ranges from 0.1–450 nN/cell [37,44]. In a recent study, the simplified model of organs, epithelial organoids, was shown to be grown in vitro with the help of embryonic and adult stem cells [45]. Broguiere et al. studied organoid-matrix interaction and fabricated hydrogels well-defined enough to support organoid generation, growth and development other than animal or tumor-derived basement membrane extract (BME) as 3D scaffolds. They found that Arg-Gly-Asp (RGD) adhesion domains on scaffolds naturally occurring on a soft fibrin/laminin matrix provided appropriate physical support. This dependency of organoid formation on RGD binding sites led to the analysis of the force distribution that organoids exerted on the matrix, which was determined by 3D-TFM. This showed that the organoid morphogenesis resulted from internal pressure along with the higher cell contractility of the differentiated cells as compared to the stem cells [46].

#### 2.1.2. Micropatterning

**Figure 5:**
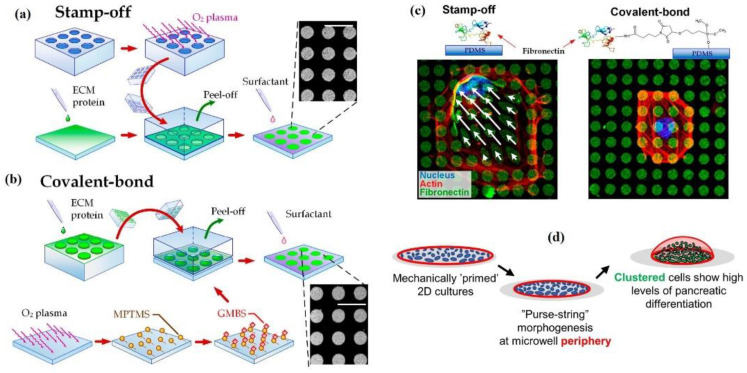
Micropatterning. (**a**) Microcontact printing (*μ*CP) of ECM proteins for achieving different protein–substrate adhesion using stamp-off based on direct adsorption and (**b**) the covalent bond based on intermediate binding molecules. Scale bars in all insets: 10 μm. (MPTMS—3-mercaptopropyltrimethoxysilan; GMBS—N-γ-maleimidobutyryloxysuccinimide ester) (**c**) ECM protein delamination in cell spreading regions on micropatterned fibronectin layers. Arrows indicate displacements of the fibronectin micro islands. Scale bar: 12 μm. (Reproduced with permission from [49]) (**d**) Proposed mechanism of action for increased PDX1 expression via collective actin “purse-string” contraction. (Reproduced with permission from [53] under Creative Commons Attribution 4.0 International License).

Micropatterning (Figure 4d) is also known as micropost, micropillar or microfabricated post-array-detectors (mPADs). It is a technique that provides a micrometer scale of a soft, 3D complex and dynamic microenvironment for both single-cell studies and the population of cells. The method is based on the basic elastic beam theory, where the calculations are based on a 1D subcellular traction force field for each micropillar displacement map [38]. This made the quantification of force much more effortless and reliable. The mechanical properties of the micropost-based substrate are tuned by varying the heights of the deformable posts without altering their surface chemistry. The micropost stiffness involves controlling cellular attachment, morphology, traction force and stem cell lineage commitment. Moreover, cell-cell and cell-ECM mechanical linkage adhesion regulate the changes in cell shape during tissue homeostasis and embryonic development. For this study, Sim et al. used ECM micropatterning and showed that the force balance in cell pairs changes with the cell spread area and aspect ratio of Madin–Darby canine kidney (MDCK) cell pairs. As a result, the constant E-cadherin molecular tension was maintained by cell pairs and total regulated forces relative to cell spread area and shape, but independently of total focal adhesion area [47]. In one of the studies understanding the fundamental material properties of actinomycin stress fibers (SFs), Kassianidou et al. have tried to demonstrate how the individual SF mechanics are related to the SF geometry and network properties in which they reside, and it is broadly understood that the inter-connection plays a vital role in cell structure and motility [48]. This was achieved by combining single-cell ECM micropatterning with subcellular laser ablation to probe the mechanics of single geometrically defined SFs. Microcontact printing (*µ*CP) is mainly used to create biomolecule patterns for the analysis of cell migration, mechanics, and tissue engineering. Here, Hu et al. characterized the coating of ECM proteins by two *µ*CP methods, i.e., stamp-off (microstructured stamps were used to create patterns by removing ECM proteins from some regions on a fibronectin-adsorbed PDMS substrate, (Figure 5a) and covalent bonds (intermediate molecules achieve covalent bonds between the protein coating and the substrate for stronger protein-substrate adhesion, (Figure 5b), and demonstrated the role of substrate-protein adhesion in cell behavior [49]. The pattern delamination can be observed on the stamp-off for micropatterned ECM protein substrates. Still, no pattern delamination was shown on the covalent bond surface (Figure 5c). This resulted in providing a new insight for micropatterning in cell-biomaterial interaction on the biointerface, indicating that insufficient adhesion by *µ*CP leads to the delamination of ECM and causes cell death. Eftekharjoo et al. proposed an in vitro experimental model and used soft PA-gels for the micropatterning of fibroblasts adherent on fibronectin lines. It can maintain fibrillar cell morphology, where the cells are allowed to interact with the specific stiffness microenvironment mechanically. This suggested that in a soft microenvironment, fibrillar cell migration does not depend on high cellular force exertion in the absence of other topological constraints [50]. In some studies, released cells have been shown to form 3D aggregates of uniform sizes when grown on small adhesive 2D micropatterned surfaces [51,52]. This proves that cells can be primed mechanically on micropatterned surfaces to form clusters, which may be sufficient to improve cell yield. Similarly, Tran et al. demonstrated control and direct cell clustering culture in adhesive micropatterns for the induction of structured pancreatic differentiation in a scalable manner [53]. The progressive transition-based F-actin (cellular actin in the form of polymeric microfilament) patterning from a uniform distribution to peripheral expression to concentration at the microwell center during the aggregation process is that a ring of contractile actin drives “purse-string” morphogenesis to compact the aggregate structures. Here, the micropatterning of cells in adherent microwells prompted clustering, increased cell density, and increased nuclear accumulation of PDX1 (Figure 5d) and NKX6.1 (earliest markers of pancreatic and beta-cell commitment, respectively, and play an essential role in the development of the pancreas towards functional insulin secretion), thus giving a more reproducible route to produce a well-differentiated pancreatic cell cluster and drive morphogenesis.

### 2.2. Measurement of ECM Mechanical Properties

The minimum strength required to detach a single-cell from its substrate is known as adhesion strength. The measurement of cell adhesion strength has created interest for many scientists in various studies, such as characterizing cancer cell stages, the compatibility of biomaterials in the human body, and the discovery of early disease diagnostics markers and drugs for disease treatments. Here, cell cultures are allowed to adhere to the matrix coated with ECM, followed by the cell adhesion detachment process where the load is applied to the adherent cells on the ECM to free them from the cell-matrix binding (Figure 2b). Thus, the applied force that is required for the detachment of a cell is quantified as the cell’s adhesion strength. For the single-cell approach, the detachment process is focused on an individual cell, and the technique used to measure the adhesion strength is either utilized for the complete detachment of a single-cell from the matrix (whole-cell detachment) or mainly focused on understanding cell adhesion kinetics by measuring the load needed to break the molecular bonds. The techniques used for the whole-body detachment are cytodetachment [25] and micropipette aspiration (MA), while single-cell force spectroscopy (SCFS; developed mainly to measure the strength of the single-cell) includes nano/micro-indentation and optical manipulation approaches that are used to perform molecular bond breakage.

#### 2.2.1. Micropipette Aspiration

**Figure 6:**
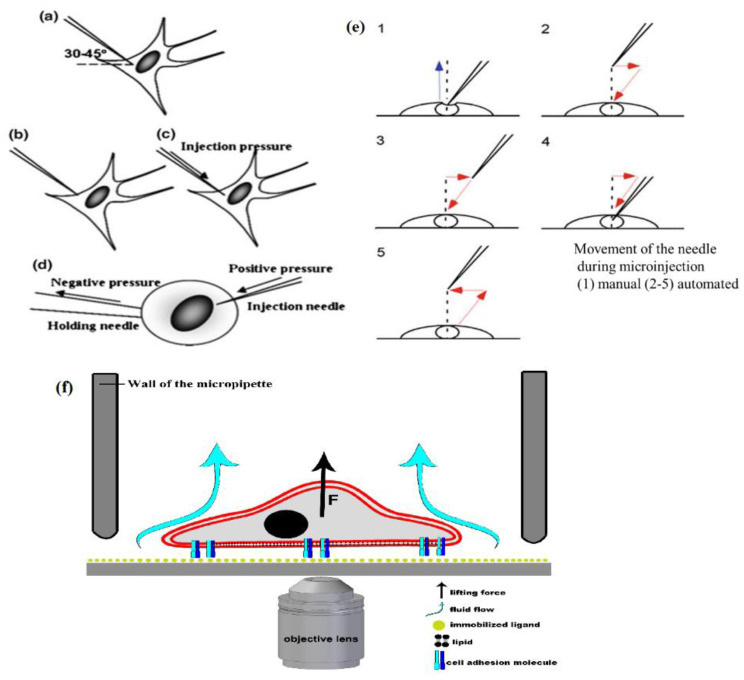
Microinjection of attached and suspended cells. (**a**) Injection needle should be placed 30–45 degrees to the bottom of the injection chamber when injecting attached cells. (**b**) The needle should be placed on the cell surface prior to penetration of the membrane. (**c**) The needle is lowered to penetrate the cell membrane into the cytosol. (**d**) A negative pressure holds the suspended cell through holding the needle and injected by an injection pressure through injection needle (Reproduced with permission from [63]). (**e**) Schematic representation of five subsequent steps of motion of the injection needle. Blue arrow depicts manual movement through the experimenter; red arrows represent automatic motions through the injection device. (Reproduced with permission from [64]). (**f**) Schematic representation of the hydrodynamic adhesion force measurement on a *single-cell* using a micropipette. (Reproduced with permission from [62] under Creative Commons Attribution 4.0 International License).

The micromanipulation techniques are usually acquired for microinjection experiments, where capillary suction is used to hold the single-cell. With the help of another capillary, the reagents are injected into the cytosolic part of the cell. The micromanipulation technique is comprised of a cell directly in contact with the probe, micro knife, microneedle, or microcapillary under the microscope [54]. The probe is now controlled by a micromanipulator, a macroscopic actuator. Micropipette aspiration (MA) is widely used for single-cell surface tension evaluation through the negative pressure measurement, and thus detaches the immobilized cell under observation via microscope. As adhered cells attach to the surface of the cultured plate or coverslip, they spread out on the surface to form discrete boundaries and shapes depending upon the cell type (Figure 6a–c). At the same time, a suspension cell is held by a holding pipette with negative pressure and an injection needle with a positive pressure performing microinjections (Figure 6d). Additionally, the movement of the needle during microinjection is shown in Figure 6e. Various mechanical properties of cells can be measured using this technique, such as investigating membrane elasticity, single-cell mechanotransduction study, molecular adhesion measurements between cell pairs, and quantifying mechanical and material properties of single-cell and cell nuclei [55]. The MA technique was used by Dufu et al., and they investigated the effect of voxelator (GBT440; anti-polymerization and anti-sickling agent) on the deformability of sickle Red blood cells (SS RBCs). This showed that the membrane shear elastic modulus of SS RBCs decreased by GBT440, concluding no change in deformability, but improved the sickle cell blood viscosity under deoxygenated conditions [56]. While Metsiou et al. studied the biomechanics of adhered and suspended breast cancer cells using the MA method, they mainly focused on comparing cell stiffness and viscoelastic parameters of MCF-7 (ER^+^) and SKBR-3 (HER2^+^) human breast cancer cell lines prior to and post-treatments of tamoxifen and trastuzumab. The mechanical parameter alterations included a remarkable increase in cell stiffness in the post-treatment of trastuzumab and changes in viscoelastic parameters in both cancer cell lines [57]. Recent studies have developed automated/robotic MA techniques, as the existing manual methods have a lack of effectiveness for cell storage and delivery, thus making MA of batch cells with low efficiency [58]. Here, Liu et al. has developed a robotic MA system capable of storing multiple cells with a feeder micropipette (FM) and picking up cells one-by-one to measure their elasticity with a measurement micropipette (MM). The key techniques involved in this system are automated cell adhesion detection, the maximum permissible tilt angle of FM, MM determination and automated cell aspiration. As compared to the existing methods, the proposed experimental system resulted in the continuous measurement of more than 20 cells with four times higher manipulation speed [59]. In another study, a single-cell methodology was reported by González-Bermúdez et al., which combined the use of MA for the measurement of living cell deformability and 3D confocal analysis of the nucleus and cytoskeleton [60]. This methodology was applied to mouse CD4^+^ T cells, which demonstrated no other geometrical or cytoskeletal features of the cell, but the size of the nucleus determined the cell deformability. With further technical advancements, MA can be combined with microfluidics technologies to have more broad applications in both single-cell mechanics studies and for diagnostics of diseases [61].

Salanki et al. studied a computer-controlled micropipette mounted onto a standard inverted microscope for probing single-cell interactions with specific macromolecules [62]. Hydrodynamic lifting force was estimated on target cells by the numerical simulations of the flow at the micropipette tip (Figure 6f). The adhesion force of surface-attached cells could be accurately probed by repeating the pick-up process with increasing vacuum applied in the pipette positioned above the cell, and this is under investigation. The automated micropipette gave higher sensitivity and less side-effects on the cells than the shear stress channel. Using this technique, the probed *single-cells* can be easily picked up and further investigated by other methods—a definite advantage of the computer-controlled micropipette.

#### 2.2.2. Single-Cell Force Spectroscopy (SCFS)—Atomic Force Microscopy (AFM)

**Figure 7:**
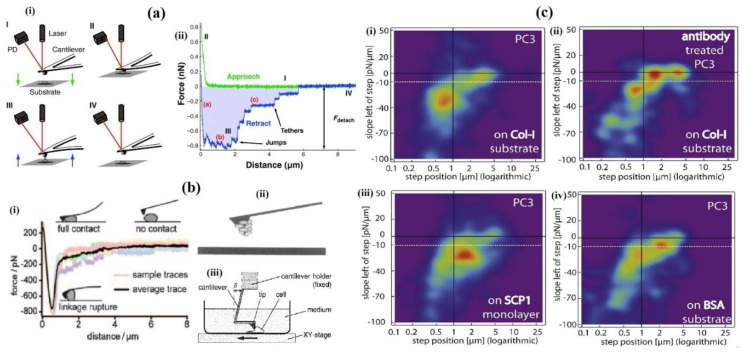
Atomic force microscopy. (**a**) Depiction of a cell-adhesion measurement (**i**) for which characteristic approach (green) and retraction (blue) traces are shown (**ii**). (**i**) In this technique, the cell and the substrate are brought into contact (aiI). The substrate that is probed can be another cell, a functionalized surface, or an organic matrix. The position on a photodiode (PD) of a laser beam (red line) that is reflected off the back of the cantilever measures the deflection of the cantilever and thus the force that acts on the cantilever. During the approach (denoted by green arrows), the cell (probe) is pressed onto the substrate until a pre-set force (usually <1 nN) is reached (aiII). After a contact time ranging from 0 to 20 min, the cell is retracted from the substrate (marked by blue arrows), and a force-distance curve is recorded (aii). This curve corresponds to a cell-adhesion signature as the strain on the cell increases. These bonds have been formed between the substrate and the cell break sequentially (aiIII) until the cell has wholly separated from the surface (aiIV). The maximum downward force exerted on the cantilever of the atomic force microscope is referred to as the detachment force (F_detach_). During the separation of the cell from the surface, two types of molecular unbinding events can occur. In the first event, the receptor remains anchored in the cell cortex and unbinds as the force increases (denoted as jumps). The second type of unbinding event occurs when receptor anchoring is lost and membrane tethers are pulled out of the cell. In the unbinding-force–distance curve, long plateaus of constant force characterize tethers. The shaded area in (**ii**) represents the measured work of cell detachment from the substrate. The lower-case letters (a, b and c) denote different phases of cell-substrate detachment. Steps I–IV shown in (**i**) are also indicated in (**ii**). (Reproduced with permission from [65]) (**b**) Atomic force microscopy (AFM) measurement of de-adhesion force. (**i**) Six sample traces for a *single-cell* are shown in shades of grey, with the average trace shown in black. At zero distance, the cell is in full contact with the cantilever, which is applying a positive force. As distance increases, the cantilever is pulled away from the glass slide surface, causing the cell–cantilever linkage to rupture and result in the zero-force, no-contact region. The force of de-adhesion was calculated as the difference between the curve minimum and the horizontal no-contact region. (**ii**) Cells-coated tips of AFM cantilever probing planar substrate. (**iii**) Principle of the measurement of a cell adhesive shear force to a material. The cell is detached from the bottom of the dish, having been applied a lateral force by the tip of the cantilever. The magnitude of the shear force applied to the cell is given as the product of the force constant of the cantilever and the deflection of the cantilever (δ). (Reproduced with permission from [68,69,70]). (**c**) Two-dimensional probability density plots of human prostate cancer (PC3) cells interacting specifically with collagen-I (Col-I) (**i**) and bone marrow-derived stem cell monolayer (SCP1) cells (**iii**) and nonspecifically with bovine serum albumin (BSA) (**iv**). (**ii**) PC3 cells treated with monoclonal antibody to CD29/integrin *β*1 also interact nonspecifically with Col-I. (Reproduced with permission from [71]).

The atomic force microscopy (AFM) technique is potentially able to measure the mechanical responses of the cells, such as modulus of elasticity, cell deformability, viscoelasticity, etc., at different loading rates. In AFM measurement of the cell, a sensitive and small probe is named cantilever, where the laser beam deflection is used to detect the nanoscopic deviations. An AFM cantilever is immobilized with an individual living cell that acts as a probe for the adhesion strength measurement between cell-cell or cell-matrix adhesions (Figure 7(ai)). After the cell adhesion process between the probe and cell or ECM coated substrate, the cantilever is withdrawn to detach the cell from its binding at a constant speed. The force-distance retrace curve of de-adhesion of a cell from a substrate is divided into three phases (Figure 7(aii)). During the first phase, the cantilever retraction force, acting on the cell, inverts from pushing to pulling. This applied detachment force is sufficiently high, due to the binding strength of the receptors together, to mechanically deform the cell cortex. The number of receptors, its binding strength as well as its geometric placement will determine the forces at which the cell starts to detach. The highest force, detachment force (F_detach_) in the force-distance curve recorded by the cantilever deflection, represents a maximum cell adhesion strength [65]. Various properties of the cell determine the detachment force, such as cortex tension, membrane properties, cell elasticity, cell geometry, as well as receptor properties such as cooperativity, placement and binding strength [66]. In the second phase, during cell-substrate detachment, either the receptors detach from the surface of the substrate, or they have pulled away from the tip of a membrane tether from the cell cortex, while its parts are still in contact with the substrate. During the final stage of detachment, the cell is no longer in connection with the substrate, where the attachment is exclusively mediated by tethers [65].

The AFM viscoelasticity measurement is performed by analyzing the reaction force when a known indentation pattern is applied to the sample and detected by the cantilever deflection. The delayed responses and the deflection amplitude provide viscous and elastic information, respectively. The 2D viscoelasticity maps are obtained when the sample surfaces are scanned. The AFM measurement outputs contain the complete information of the viscoelasticity coefficient with a high spatial resolution (nm). As the setup and scanning are time-consuming, the technique has low throughput. The cantilever applies localized deviation in a nanoscopic length scale; thus, only nanoscopic viscoelasticity measurement is accessible [67]. Generally, the AFM technique is used in three different ways to measure cell adhesion force. In the first method (Figure 7(bi)), the cell surface is touched by the modified cantilever tip, and the adhesion force is a measure between the tip and the cell [68]. The second method (Figure 7(bii)) is to measure the force between the cell and the surface, where a cell is fixed at the tip of the cantilever [69]. In comparison, the third method (Figure 7(biii)) is to measure the adhesion force by applying a lateral shear force to the cell until the cell detaches from the substrate surface [70]. However, the SCFS-AFM probe can be studied for various adhesion aspects without any restriction on the cell type or cell adhesion molecules used.

Sariisik et al. used the AFM technique to evaluate the detachment steps of long membrane tethers to be distinguished from shorter jump-like force steps, which are typical for cytoskeleton-anchored bonds [71]. The AFM sensor was anchored with an immobilized prostate cancer cell line (PC3) and allowed to interact with three different substrates—bovine serum albumin (BSA), collagen-I (Col-I) and bone marrow-derived stem cell monolayer (SCP1). The plots of probability density revealed the β1-integrin specific interactions anchored to the cytoskeleton, while the non-specific interactions are mainly membrane-anchored, thus giving a better understanding of receptor-ligand interactions (Figure 7(ci–civ)). Zhang et al. showed the combination of SCFS with electrochemical-AFM to quantify adhesion between live single-cell and conducting polymers whilst simultaneously applying a voltage to switch the polymer from oxidized to reduced states electrically. This represents non-specific interactions where single-cell adhesion increases, as the polymer is in a fully reduced state, indicating stronger cell binding to sulfonate groups as compared to hydrophobic groups. Dragovich et al. studied the role of endothelial surface glycocalyx (ESG) in the adhesion force between human umbilical vein endothelial cells (HUVECs) and leukocytes under normal or inflammatory conditions using AFM based single-cell assay [72]. Detachment force and work were measured for every separate event. The results demonstrated the dual function of the ESG layer, i.e., it prevents the adhesion of leukocytes on resting endothelium, while under inflammatory conditions, it participates in leukocyte-endothelial interaction with molecules such as selectins. Physical properties (i.e., roughness and hydrophobicity/hydrophilicity) of the substrate have shown a significant impact on the required cell adhesion force. The effects of physical properties on 1,4-polyisoprene (PI) and polyethylene oxide (PEO) thin films on the adhesion of two melanoma cell lines, the primary tumor site from the vertical growth phase (VGP WM115) and from its metastasis to the skin (WM266-4) were studied using SCFS-AFM. The results showed better adaptability of metastatic WM266-4 cells to different substrate properties in comparison to VGP WM115 cells [73]. Zhang et al. used the AFM-based SCFS technique to quantify the required detachment force for a single fibroblast cell in contact with the conducting polymer, polypyrrole doped with dodecylbenzene [74]. Their study suggested that the de-adhesion force is due to the unbinding of α5β1 integrin with surface absorbed fibronectin using the blocking experiment of antibodies. Chièze et al. developed a method to characterize single-cell adhesion properties by using a protein-functionalized AFM probe, which can be proved to be effective in cancer cell migratory abilities [75]. Liebsch and Schillers developed the SCFS-AFM approach to quantify the heparin effect (anticoagulant, suppresses cloak’s formation by inhibiting the interaction between P-selectin, i.e., cell adhesion molecule, with its ligands on cancer cells, thus reducing the rate of metastasis) and adhesion force between the platelets and human non-small lung cancer cells (A-549). The study showed that heparin’s anti-metastatic effect blocks the P-selectin, which indicates a maximum decrease in the adhesion force [76]. In severe irreversible damaged muscle cells, cell therapies are still limited to treat the injuries. Ribeiro et al. investigated the effect of the surface charge of piezoelectric polyvinylidene fluoride (PVDF; material that offers new opportunities for skeletal muscle tissue engineering) on C2C12 myoblast cell adhesion [77]. This interaction was studied using the AFM technique, measuring the higher cell adhesion force of individual cells on PVDF samples with overall negative surface charge, which indicated that the surface charge/polarization is an important parameter affecting cell adhesion to determine the cell fate and its behavior.

#### 2.2.3. Single-Cell Force Spectroscopy (SCFS)—Biomembrane Force Probe (BFP)

**Figure 8:**
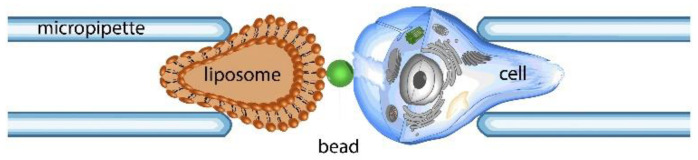
Schematic illustration of a biomembrane force probe. A liposome is sucked into a micropipette that is biochemically glued to a latex bead. By pushing or pulling the liposome, a force can be exerted on the bond between the cell and the bead. As the latter can be coated with a protein of interest, receptor-ligand bonds can be studied under a controllable loading rate and force. (Reproduced with permission from [18]).

The quantification of single-molecular bonds has been carried out by using a biomembrane force probe (BFP) (Figure 8), a multifaceted tool used in a wide force range (0.1 pN–1 nN) and loading rates (10^6^ pN/s) [78]. This technique uses a force transducer made of a biotinylated liposome or erythrocyte (with known stiffness) held by a micropipette with suction pressure. A latex microbead was attached to the erythrocyte or vesicle; this assembly constitutes a powerful nanodynamometer and is used as a force transducer [79]. When this probe is in contact with the targeted cell, bond formation occurs between them, followed by a detachment process where the targeted cell is pulled away using the piezoelectric manipulator. Chen et al. characterized the conformational changes—bending and unbending of single αVβ3 integrins (which play an essential role in phagocytosis, angiogenesis, tumor metastasis and hemostasis)—using the BFP technique [80]. Thus, between two conformations, the speeds and rates of conformational transitions, the probability of conformational changes and dynamic equilibrium were regulated by tensile force, dependent on the ligand and altered by point mutations. In another work, a DNA origami tension sensor was described that maps the pN forces generated by living cells. Single-molecule force spectroscopy was used to determine the probe tension response thresholds. Further, computational modelling showed that hairpin unfolding is semi-cooperative and orientation-dependent [81]. Chen et al. demonstrated the measurement of the dissociation of single-bonds using three receptor-ligand systems, having an essential function in vascular and immune systems using live-cell dynamic force spectroscopy (DFS), BFP. The transition of bonds between two dissociation states achieved a disruptive effect, showing that faster force loading induces more bonds to adopt the fast-dissociating form. This new biophysical model took into account the effects of both the loading rate and force magnitude [82].

#### 2.2.4. Single-Cell Force Spectroscopy (SCFS)—Optical Tweezers (OT)

**Figure 9:**
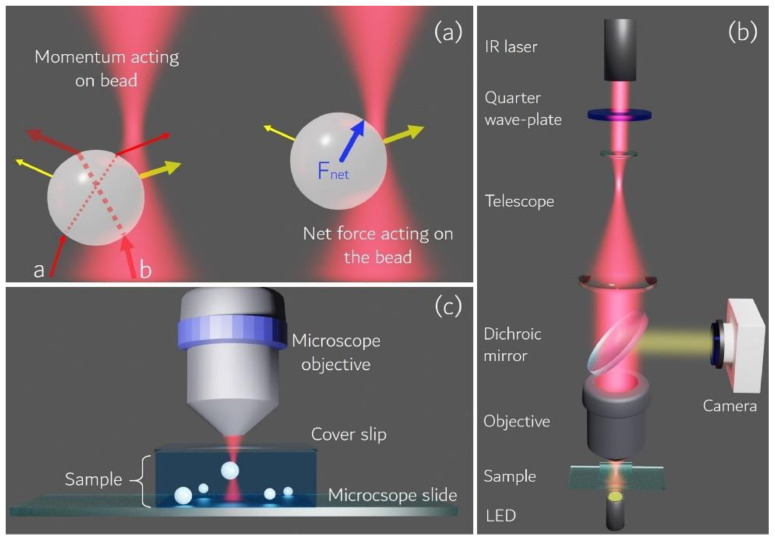
Optical tweezers. (**a**) An intuitive representation of the trapping process in the Mie regime. The momentum (yellow arrows) of two rays, *a* and *b* (red arrows), with different intensities propagating through a sphere is shown. The blue arrow indicates the restoring net force drawing the particle into the focal region. (**b**) Schematic of conventional optical tweezers (OT) setup—The optical trap is formed inside a sample chamber with an objective with high magnification and numerical aperture. The trapping process is imaged with a camera. (LED—Light emitting diode) (**c**) Model of the trapping process inside the sample chamber. (Reproduced with permission from [88] under Creative Commons Attribution 4.0 International License).

In this method with optical tweezers (OT), the probe with the small particle is attached to the cell surface. Here, particles are made of polystyrene or silica with a high refractive index. The radiation pressure of a highly focused laser beam is used to trap and manipulate microscopic and neutral objects—a technique developed by Arthur Ashkin and co-workers in 1986 (Nobel Prize in 2018). These small dielectric spherical particles experience two kinds of forces, namely scattering and gradient forces [83]. The cell struck by the photons along with their propagation direction is known to be the scattering force, while the field intensity gradient produces gradient force. These forces exerted on the particle depend on the particle size (r) and laser beam wavelength (λ), and the ratio between them defines the relevant physical phenomenon behind the trapping process. For example, when the r >> λ, the process is described in the frame of Mie regime as shown in Figure 9, where the particle is transparent for the light beam and behaves as a lens focusing the incident light [84]. OT vastly contributed to the study of micro and nano-objects in biology and physics. It has been proven to be a safe and non-invasive manipulation for living objects such as biomolecules, bacteria, viruses and cells [85,86,87]. The advantages of OT over AFM and micropipette aspiration are higher sensitivity, no contamination, whole-cell control in a non-destructive manner and controllable laser light intensity to adjust the applied force with a pN accuracy [88]. Individually, OT is a great instrument, but when combined with other modalities such as fluorescent microscopy [89], microfluidics [90] and Raman spectroscopy [91], etc., it has enhanced its potentiality for further cellular analysis.

Many types of research have been carried out, where OT approaches are directed to study the intrinsic properties of RBCs such as viscosity, deformability and cell-cell interactions such as adhesion and aggregation. Additionally, experimental implementations include the trapping of whole-cell, for optical stretchers, sorting and rotators or force exerted through microbeads attached to the membrane. Steffen et al. used holographic OT and SCFS for the study of the active participation of RBCs in thrombus solidification, where the increased levels of internal Ca^2+^ concentration induced irreversible intercellular adhesion of RBCs, similar to lysophosphatidic acid (LPA) stimulation, a compound released by activated platelets with an adhesion force of approximately 100 pN [92]. OT is also useful in the detection of healthy and pathological cells, which can be applied in clinical diagnostics. Moreover, OT was used to study the effect of doxorubicin (a commonly used anticancer drug) on the RBCs, DNA and leukemic blast cells by stretching them with 4.5 µm beads attached to the opposite sides of the cell [93]. The study showed an increase in cell elasticity and a double decrease in the stiffness of healthy RBCs and death of leukemic blast cells due to the high toxicity of doxorubicin. The incorporation of the drug into the cell membrane was confirmed by confocal laser scanning microscopy (CLSM), and thus the distribution of doxorubicin over the entire cell volume. Many such applications of OT have been carried out, such as blood cell studies, adhesion, cell interactions, etc. [88]. In another study, Gou et al. has reported a case study where optical traps were used at various positions of the bone marrow stromal cell layer to gather leukemia cells, thus enabling their interactions and maintaining the cell contact for a few minutes by applying a small trapping force [94]. The results showed that cell adhesion and interaction were affected by the amount of cell adhesion molecules present on the cell surface. Additionally, specific chemokines are responsible for the migration of the leukemia cells, their adhesion state and the triggering of activation for the cell-cell interaction. In another study under physioxia conditions, the OT based assay was used to evaluate single diffuse large B-cell lymphoma (DLBCL) cell adhesion to mesenchymal stromal cells and Matrigel, and also to explore the role of integrins in single lymphoma cell adhesion. The study resulted in reducing adhesive properties under physioxia between the lymphoma cell lines and both stromal cells and Matrigel. It also showed that under physioxia conditions, β1, β2 integrins and cadherin-2 expression are impaired, thus reducing lymphocyte adhesion [95]. In work reported by Vaibavi et al., the effect of calcium channel blockers was studied on the viscoelastic properties of MCF-7 and the vascular endothelial (HUVEC) cell line. Here, OT was used to measure the effect of drugs on the mechanical properties of the cytoskeleton and membrane, showing a significant effect of the clinically approved drugs on the circulating tumor cells in the blood [96]. The DNA, RNA and proteins from a dynamic complex with other proteins resulted in a conformational change of protein that is key to function. Here, Avellaneda et al. combined OT and fluorescence imaging to monitor the binding and conformational change of individual proteins by detecting the tethered fluorescent protein’s emission and a molecular chaperone complexed with its client [97].

#### 2.2.5. Microfluidics

Microfluidics and lab-on-a-chip technologies have represented a revolution in different laboratory experimentation. In the last two decades, the basic microfluidics techniques have been used for different cellular analysis such as cell separation, cell manipulation, cell lysis and cell culture. Moreover, microfluidics has been widely applied based on cell handling techniques in various fields of immunoassays, stem cell research, neuronal cells, polymerase chain reaction (PCR), organ-on-chip and the identification and analysis of circulating tumor cells (CTCs) [98]. Thus, it has been proposed to be a high-throughput screening technology because of its properties of miniaturization, integration, automation, low-cost analysis, easy handling and low sample consumptions (nl volumes of liquids), and it is suitable for cell-based assays. The reduced device size and small sample volumes lead to greater efficiency of the device, portability of the instrument and quick read-out data, thereby allowing for fast sampling times and disposability. Advanced microfluidic techniques have been used for single-cell analysis, especially for the measurement of cellular shape, size and deformability [99]. They are studied and reviewed from the viewpoint of two methods based on the controlled flow—the passive microfluidics drives a constant flow within the microchannel and has been widely used for deformability tests. At the same time, active microfluidics can control flow and suspended objects in real-time by combining mechatronics and robotics with microscopic biological materials. With this ability of real-time observation, cell adhesion studies have been made easy. The devices are constructed of optically transparent PDMS material bonded on glass using a soft lithography rapid prototyping process. The optical transparency enables the use of various real-time microscopy techniques to evaluate cell behavior under varying experimental conditions [25]. A microfluidic channel was used to measure cell adhesion strength by a detachment of controllable load adhered on a single-cell, resulting in the investigation of the cell geometry effect on adhesion strength [100]. The technology has grown and upgraded from the study of cell adhesion properties and dynamics into the development of tissue-on-a-chip and later into organ-on-a-chip for biochemical and pathological studies. The monolayer of cells was grown in the microfluidic channel to mimic the human vascular system, and it can be used for bioengineering and biochemical analysis [101]. Basabe-Desmonts et al. reported a simple method, interfacial platelet cytometry (iPC) platform with a self-powered microfluidic device enabling platelet separation, and its adhesion studies [102]. To understand the mechanism of metastasis, CTC adhesion studies are needed. In one of the studies by Mao et al., the microfluidic method was used to analyze the adhesion strength of the CTCs attached to the endothelial cells (ECs) at the single-cell resolution [103]. The microfluidic device was supplied with the trypsin zone to measure the detachment adhesion strength by the time required. The results indicated different adhesion strength by different CTCs, and the cells that hold strong adhesion ability towards the HUVEC cell layer would show stronger invasiveness. The extremely rare CTC detection from the blood would be of great interest to diagnose cancer at early stages. Cho et al. employed a 3D microfluidic device in the detection and characterization of CTCs using the immunofluorescence staining process. The study reported the rapid selection of live CTCs from the whole blood on a self-assembled cell array (SACA) chip for further characterization using automatic imaging and an in situ cell capturing process. With 1/180 recognition sensitivity, the entire process takes 120 min for the analysis of 4 mL of the whole blood sample, along with the high viability after the rapid selection process [104]. In another study, the microfluidic platform with biosensing applications using gold surface and epithelial cell adhesion molecule (EpCAM) antibody (Ab) was used for the detection of human breast cancer cell line (MCF-7). The EpCAM Abs immobilized on self-assembled monolayers (SAMs) of various molecules with different functional groups were formed on a plain gold surface to achieve maximum and specific CTC-antibody interaction. The cell attachment events were monitored by using a fluorescent microscope in terms of the number of captured cells. This method with the antibody immobilization technique can be used for CTC detection and capture using a microfluidic biosensor device [105]. Elias et al. have used a microfluidic platform combined with an on-chip micropipette aspiration to study the physicochemical environment for the mechanics of cell membrane by the deformation of giant unilamellar vesicles (GUVs) [106].

## 3. Adhesion Force Measurement for Single-Cell Microorganisms

**Figure 10:**
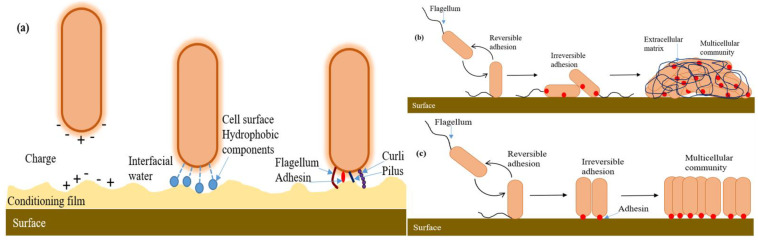
Biofilm formation of the microorganism. (**a**) First interactions between the surface and the bacterium. Once the bacterium is close to the surface, the heterogeneity of both the surface and the bacterium at the microscale level affects the adhesion process. Although the bacterial cell is usually negatively charged, the cell surface is highly heterogeneous, exhibiting different charges around the cell body. In addition, the presence of the conditioning film (orange) can modify the physicochemical properties of the solid surface (by altering charge, potential and surface tension) and thus affect local adhesion. At the nanoscale level, the thin layer of water (interfacial water, light blue) present on the surface can potentially be a barrier to cell adhesion. Hydrophobic components on the cell surface (dark blue), such as proteins, the polymeric brush layer and extracellular polysaccharides, can displace interfacial water between the bacterium and the surface and enhance hydrophobic interactions, thereby promoting close contact between the bacterium and the surface. Once the bacterium is sufficiently close to the surface (<1 nm), adhesins (red) and bacterial cell appendages (such as flagella (brown), pili (blue) and curli (purple)) can interact with the solid surface and have direct or indirect roles in adhesion. (**b**) For some bacteria, such as *Pseudomonas fluorescens* or *Escherichia coli*, initial surface contact is mediated by flagella and pili, which leads to polar adhesion. These bacteria transition from reversible to irreversible adhesion by repositioning the cell body to a longitudinal position. The adhesion is enhanced by the synthesis of protein or polysaccharide adhesins (LapA for *P. fluorescens* (red) or Pel for *E. coli*). Irreversible adhesion leads to the formation of a multicellular community embedded in an extracellular matrix composed of polysaccharides, proteins and DNA. (**c**) Bacteria, such as *Caulobacter crescentus* or *Agrobacterium tumefaciens*, initiate surface attachment through the polar flagellum. Cells stay attached via their pole and achieve permanent adhesion through the secretion of a polar adhesin (red dots: holdfast for *C. crescentus* and UPP for *A. tumefaciens*). Consequently, the incipient multicellular community is composed of cells mainly oriented polarly and is devoid of an extracellular matrix. For simplicity, the presence of a sinusoidal flagellum illustrates the motility of free-swimming bacteria, and the flagellum is the only external appendage shown. (Figure redrawn from [129]).

The pathogenicity of the microorganism strongly depends on the multicellular generation assemblies known as a biofilm. The biofilm grows due to the bacterial attachment that occurs on all wet surfaces and is undesirable to be formed on human-made surfaces because of infections in the biomaterials, food contamination, plugging or corrosion of the pipeline, etc. Bacterial cell colonization is responsible for environmental change such as chemical composition [123], compliance [124], wettability [125] and nano topography [126]. Biofilm formation consists of the critical step of the surface attachment of a single bacterial cell, which is mediated by adhesins and extracellular polymeric substances (EPS) such as DNA, proteins, lipids, oligopeptides, and lipopolysaccharides. As shown in Figure 10a, the host-bacteria adaptation mechanism often depends on the extracellular components, which facilitate adhesion between approaching surfaces by reducing their energy barrier [127]. The EPS secretion accumulates eventually after the initial cell attachment, leading to changes in biofilm elasticity and stiffness. Initially, the proliferation of bacteria within the monolayer depends upon the level of confinement. Then, just after a few division cycles, they form microcolonies and become 3D. The physics behind the morphogenesis of microcolonies involves complex mechanical couplings between adhesion, friction, cell elongation, cell rearrangement and steric interactions (Figure 10b,c) [128,129]. The bacteria adhere to the surface during monolayer expansion; after that, bacteria build up turgor pressure during the cell elongation. This pressure is massive compared to the substrate-bacteria adhesion force, resulting in the detachment of neighboring cells and even their ruptured adhesion [130]. Hence, it is still unclear how they dynamically and spatially coordinate between cell elongation and adhesion forces to maintain surface-attached communities. However, biofilm formation is also essential in bioremediation, waste-water treatment, and symbiosis to form the internal microbiome of an organism. Thus, it is vital to probe EPS-mediated adhesion and quantify the *single-cell*-surface interaction mechanism, which may help to lead new strategies to gain insights into the biofilm such as in removal strategies, antifouling surfaces or in agriculture and health care [131].

Eukaryotic cell or bacteria (single-body) detection is essential and required in a large number of sectors, such as environment monitoring, food safety, biomedicine, and pharmaceutical industries. While keeping minimum disruption of the measuring system, the risk of contamination should be kept low by using contactless detection techniques. The single-body detection of bacteria in food, drinking water, and bio-fluids (e.g., blood and urine) would help us in the early diagnosis of various infections and, hence, early-stage treatments with increased success. Moreover, single-cell detection would be of great help in the diagnosis of candidiasis (systemic fungal infection caused by *Candida albicans*) or to detect the presence of CTCs in the bloodstream.

Zeng et al. have fabricated a single-cell probe using a tipless AFM cantilever coated with cell adhesive Cell-Tak to measure the single bacterial cell adhesion force of *S. xylosus, S. epidermidis, P. fluorescens* and *E. coli* on different surfaces. The single-cell probe offers controlled and stable cell immobilization during the AFM force measurement and thus shows a significant advantage over the commonly used multi-cell probe [132]. *Xylella fastidiosa*, an economically significant phytopathogen, was analyzed by Janissen et al. at single-cell resolution using the nanometer-resolution spectro-microscopy technique, and they determined the adhesion role of various EPS at each stage of its life cycle [133]. The unspecific electrostatic interactions are the cause for single-cell adhesion at the cell polar regions, suggesting that EPS accumulation is needed for firm attachment and irreversibly adhered cells.

**Figure 11:**
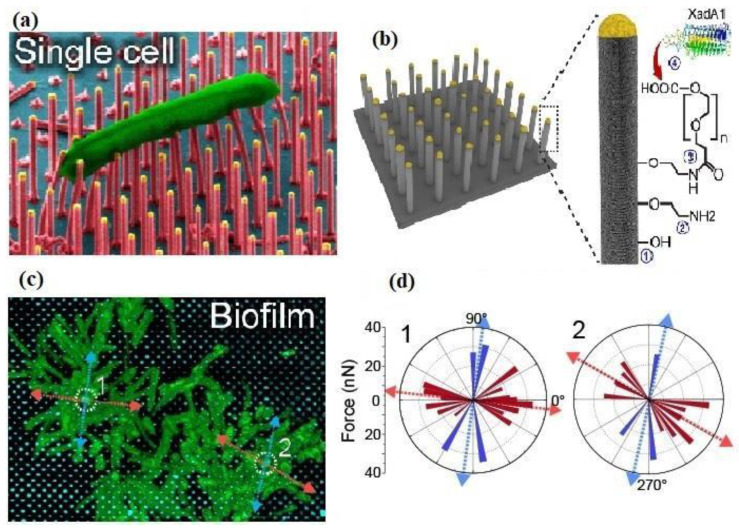
Adhesion force for anchoring a single-cell to a biofilm. (**a**) Image showing the deformation of nanowires in direct contact with the attached cell body (colorized in green). (**b**) Schematic representation of the functionalization protocol for the indium phosphide (InP) nanowire array using polyethylene glycol (PEG) cross-linker and XadA1 adhesin. (**c**) Confocal laser scanning fluorescence microscopy (CLSM) image of the small biofilm, with the marked position of the two nanowires and the average directions shown in c. (**d**) The magnitude and direction of the forces calculated for the two nanowires with arrows indicating the predominant, average direction of movement; the noise was excluded by only considering forces with values larger than control +10%. (Reproduced with permission from [134] under Copyright (2016) American Chemical Society).

Sahoo et al. fabricated indium phosphide (InP) nanowire arrays to study the early stage of biofilm formation of the phytopathogen *X. fastidiosa*. The ex vivo studies measure up to 45 nN single-cell adhesion forces, mainly depending on the orientation of the cell with respect to the surface. The cell poles consisted of larger adhesion forces along with the filaments and EPS layer for additional mechanical support. As shown in Figure 11a, the XadA1 adhesin-coated surface demonstrated an enhancement of adhesion force for anchoring a single-cell to a biofilm, which indicates the specificity by the bacterial pathogen to the host tissue. Figure 11b depicts XadA1 adhesion protein immobilized on both flat and nanowire array surfaces. Additionally, the results were used to plot the magnitude and directions of the measured adhesion forces (Figure 11d). These directions are also indicated in Figure 11c, showing the top CLSM image of the small biofilm [134]. In work presented by Wang et al., silicon nanowire array grafted with poly 2-dimethylamino ethyl methacrylate (pDMAEMA) by surface-initiated atom transfer radical polymerization (SI-ATRP) was used to show the fine control of protein adsorption and bacterial attachment on the modified material by tuning the wettability of the surface via varying pH and NaCl concentration in the environment. The potential applications of this approach were in biosensors, environmental treatments using microbes and in the controlled adsorption and release of the drug [135]. The silicon nanowire array modified with quaternized pDMAEMA using benzyl chloride was reported to be a high density and extremely useful antibacterial polymer, resulting in high bacterial adhesion and killing [136]. Moreover, Rizzello et al. demonstrated that a pure physical stimulus by nanoscale variation in surface topography plays an essential role in analyzing the morphological, genetic and proteomic profile of bacteria [126]. It was observed using AFM and SEM that the type-1 fimbriae which disappeared from *E. coli* adhered onto the nanostructured substrates. Thus, from the bacteria onto the flat gold surfaces. This suggests that the molecular mechanism of microbes adhering to surfaces is of significance, which will help to design novel biomaterials with active biological functionalities.

**Figure 12:**
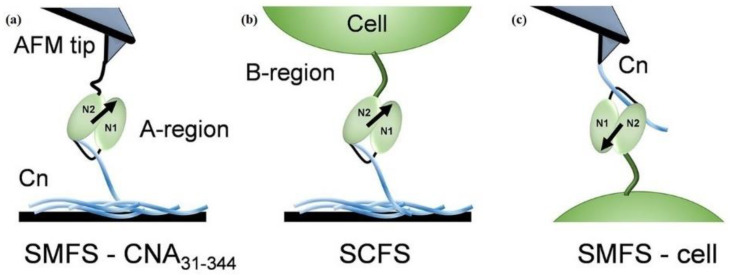
Atomic force microscopy analysis of the Cna-Cn interaction in vitro and in vivo. (**a**) Single-molecule force spectroscopy (SMFS) of recombinant CNA_31–344_, i.e., a portion of the A region that contains the ligand-binding domains. Single-cell force spectroscopy (SCFS; **b**) and SMFS (**c**) of full-length Cna expressed in *S. aureus* Phillips bacteria (for the sake of clarity, the N3 domain is not shown in the middle and right cartoons). As illustrated, Cna binds to Cn via the collagen hug mechanism, where the N1N2 subdomains cooperate to wrap around the rope-like structure of collagen. (Reproduced with permission from [137] under Creative Commons Attribution 4.0 International License).

In another study by Herman-Bausier et al., AFM has been used to measure the mechanical strength between the *S. aureus* cell surface adhesion protein (Cna) and the host extracellular matrix protein (collagen Cn-binding protein). They demonstrated that the Cna-Cn bond in vivo held ~1.2 nN strength, reflecting a high-affinity binding by the “collagen hug” mechanism. It also showed that the previously unknown mechanism of the B region of Cna functions as a spring, capable of sustaining high forces and helping the A region to maintain its ligand binding bacterial adhesion (Figure 12). The authors also studied and quantified the antiadhesion activity of monoclonal antibodies (MAbs) against Cna, suggesting an efficient blocking of single-cell *S. aureus* adhesion [137].

Duvernoy et al. have monitored single-cell adhesion during microcolony formation using laser ablation to track successive divisions while preventing steric interactions between daughter cells by ablating one of the two daughter cells after each division, resulting in the adhesion forces of rod-shaped *E. coli* and *P. aeruginosa* being polar. Further, the substrate adhesion during colony expansion was monitored using AFM and polyacrylamide Traction force microscopy (PA-TFM), where embedded fluorescent beads served as deformation markers. The adhesion stress generated was calculated by mechanical tensions in the growing colony, finding that the adhesive stress is heterogeneous and dynamic. The adhesion foci force depends on the number and nature of the adhesive links engaged in the cell-surface interactions [138]. Wolf et al. presented differences in the biofilm colonization process on different materials, such as indium tin oxide (ITO; the material used for the impedance sensor construction to measure single-cell adhesion), polypropylene, polybutylene, polyethylene and polyvinyl chloride (used for water supply system construction) by using AFM and SEM techniques. The study presented the use of ITO for the construction of a water supply system and sensor material used to measure biofilm development [139]. The MA force spectroscopy technique was used to measure in vivo single-cell adhesion forces and to reveal that the flagella of microalga provide light-switchable reversible adhesive contact with the surface, occurring within seconds. The study resulted in showing the natural functionality of the microalgal adhesiveness to regulate the transition between the surface-associated state and planktonic. This helps in adaptation and to optimize the photosynthetic efficiency in coexistence with phototaxis [140]. Byvalov et al. investigated the quantitative role at the initial state of bacterium adhesion of lipopolysaccharides (LPS) from the outer bacterial membrane to the host cell membrane, employing OT under the “object shadow” conditions. The optical trap comprised of J774 macrophage being approached by LPS-coated microsphere to initiate their binding and then rupturing the bond by moving back, resulting in the rate of detachment of 3–6 pN/s [141]. Antimicrobial peptides (AMPs) have shown promising effects to fight against bacterial infections, given their broad-spectrum activity and rapid bactericidal action [142]. Moreover, they have been found to be active against viruses, fungi and cancer cells [143]. Thus, aiming to develop AMP-based pharmaceuticals, a complete understanding of AMP-membrane interaction is needed. Mescola et al. studied the effect of magainin H2 (Mag H2) AMPs provided with a higher degree of hydrophobicity on giant unilamellar vesicles (GUVs). They mainly concentrate their impact on lipid bilayer mechanical properties and permeabilization activity by using the MA technique and flickering spectroscopy. The results showed that, as the hydrophobicity of Mag H2 increased, it affected its selective conferring, notwithstanding the enhanced hydrophobicity, and it had a strong permeabilization activity on zwitterionic lipid bilayers [144]. In recent studies, nanometer scale-controlled surface structuring has proven to be a useful strategy to provide antimicrobial properties of different materials. Here, a study presented by Dauben et al. has successfully quantified the cell adhesion data between immobilized gold nanoparticles (AuNPs) on gold thin films and *C. albicans* (fungus associated with implant infections) with controlled nano-contact point density performed by using SCFS. The force-distance curve analysis revealed that the adhesion force required for *C. albicans* cells from the surface is about ten times lower on an AuNP structured surface compared to unstructured surfaces, thus enhancing antimicrobial properties [145].

## 4. Single-Molecule Adhesion Force

While studying mechanical forces, it is necessary to classify the interactions at either a cellular or molecular level to determine the approach and methods that can be used in obtaining information on a sensitive mechanical system. Cellular interactions involve nN range forces resulting from the application of force by whole-cell on the environment. This helps us to understand the interactions between cells and valuable information obtained to study the cell behavior and morphology affected by the forces. At molecular level interactions, the information is gained regarding conformational changes due to forces and how this mechanotransduction pathway is triggered. In a study reported by Chowdhury et al., the power of molecular forces shows notch receptor activation dependent on the energy required between 4 and 12 pN [146]. Thus, a considerable variation in force magnitude is being studied when compared between cellular and molecular forces.

The macromolecules that cause structural changes of the cell under the influence of the mechanical force can be directly probed using the single-molecule force spectroscopy (SMFS) technique. The insights from the SMFS experiments can make us understand the fundamentally important molecular mechanisms with the help of a *single-cell*, which can sense, transduce, and generate mechanical forces in vivo. SMFS can be probed into both mechanical and non-mechanical proteins on cell membranes to provide insights into their functionality. Researchers have taken an interest in a concept called free energy landscape (theoretical space, where a protein molecule diffuses and samples different conformations) [147] by applying mechanical forces, where the molecule is forced to sample conformations in an accelerated manner along a specific reaction coordinate. This allows us to observe and quantify discrete states of a molecule that may be biologically relevant but transient in the absence of force. The experimental apparatus commonly used for the SMFS experiments include centrifuge force microscopy, acoustic force microscopy, AFM, optical or magnetic tweezers, and BFP [148]. SMFS can study various biological systems where mechanical forces play an essential role. For example, cell adhesion [149,150], ECM [151,152], blood coagulation [153,154], muscles [155,156], hearing [157,158], DNA/RNA molecular motors [159,160], protein folding at the exit tunnel of the ribosome [161,162], protein unfolding and proteolysis by the proteasome [163,164], and many more. Single-molecule AFM (smAFM) and steered molecular dynamics (SMD) were used to elucidate the response of contactin-4 (CNTN-4; cell adhesion molecules—CAM localized at the neuronal membrane, a key role in maintaining mechanical integrity and signaling properties of the synapse) protein to mechanical stress. The robot-enhanced AFM technique help us to understand the existence of weak interactions stabilized between domains and provides insights into the nanomechanics of a multidomain protein [165]. Zhang et al. employed the in vitro SMFS technique along with confocal microscopy to study the modulation effect of the cAMP-PKA-dependent pathway on intracellular adhesion molecule-4 (ICAM-4; mediator of abnormal adhesion between RBCs and endothelial cells, appears on the RBC membrane and binds to receptor αvβ3) activation [166]. The study showed that the unbinding force between ICAMP-4 and αvβ3 for normal and sickle cell RBCs remained the same. In a study by Burgos-Bravo et al., OT was used as a single-molecule force transducer along with the Dudko–Hummer–Szabo model to calculate the kinetics of Thy-1/αvβ3 (integrin mediated bidirectional cell-cell communication between neurons and astrocytes) dissociation [167]. The OT with fluorescence imaging was used to monitor the conformational changes of individual protein in protein-DNA coupling. This technique provides 40 times higher coupling yield as compared to the existing ones [97].

SMFS has also enabled the functional analysis and imaging of individual receptors on the surface of the pathogen. SMFS helps to capture the binding force and dynamics of single adhesins. In the past few years, the most striking discovery is the Staphylococcal adhesins, i.e., SdrG, ClfA, and ClfB, bind to their protein-ligand with ~2 nN strong forces, equivalent to the covalent bonds. Thus, pathogens have developed much stronger bonds as compared to the known prototypical streptavidin-biotin bond (strongest in nature, ~0.2 nN) to sustain pathogenicity [168]. Herman et al. used both SCFS and SMFS and found that SdrG adhesin binds Fg, reflecting the DLL (dock, lock and latch) mechanism with a 2 nN force [169]. Harimawan and Ting used the SMFS-mediated AFM technique to probe the adhesive nature of EPS produced by *B. subtilis* and *P. aeruginosa*. The comparison indicated that the presence of polysaccharides promoted EPS adhesion strength, while a minimum adherence effect was observed in the case of proteins. Thus, enhanced cell adhesion leads to the growth of biofilm [170]. Another study of interest has shown the role of the adhesion forces of bacterial appendages, e.g., pili, which is incriminated in the virulence of the pathogen, as they can resist mechanical stress applied by the mucus or the blood flow in the human body. In a study by Becke et al., the binding mechanism of pili adhesions, such as RrgA and RrgB of *S. pneumoniae* to fibronectin (Fn) and collagen (Cn), respectively, was demonstrated [171]. In another study by Rivas-Pardo et al., a high mechanostability was linked to many adhesins to the Spy0128 pilus mediated adhesion of *S. pyogenes* [172]. Still, the techniques used currently remain niche even though there is a high-potential for SMFS; however, it has not been widely adopted by the molecular biosciences community.

## 5. Recent Technologies for Single-Cell Adhesion Study

### 5.1. Environment Scanning Electron Microscopy (E-SEM)

Cell-cell and cell-substrate adhesion are the significant characteristics of tissue engineering. As compared to the analysis of cell activities, i.e., growth, proliferation and differentiation on the artificial scaffolds, the study of single-cell adhesion is a bit of a challenge, and thus it can be quantitatively performed via force measurement. Several available force measurement techniques are challenging to implement directly when dealing with cell adhesion. The results given by some of the conventional methods, such as shear force and centrifugation assays, are inexact, insensitive, and not quantitative. OT has a relatively low force resolution of about a few hundred pN, which is found to be inadequate for the cell adhesion force measurement (expected to be in the range of µN). The micropipette approach is useful for measuring the suction force on the cell surface. Still, it lacks the ability to measure the adhesion force or the releasing force of the cell from the substrate. The microgripper has the capability to pick up the cell, but is unable to calculate the adhesion or releasing force simultaneously [173,174].

Generally, AFM is used in three different ways to measure cell adhesion force, but it becomes difficult to apply these techniques to every study. A few of the demerits observed in these techniques are that it becomes challenging to identify the part of the cell that interacts with the cantilever tip [68], and secondly, due to the narrow end of the cantilever, it becomes difficult to assemble the cell at the tip and also the chemical glue used to fix the cell at the tip may affect the cell viability [69]. The sharp tip of the cantilever may damage the cell while applying a lateral shear force to the cell until the cell detaches from the substrate surface. Some researchers also use cytodetachment or the back-end of the cantilever, but AFM can obtain results and images only after the tip finishes the scanning of the sample surface [175]. The AFM system is restricted to micrometers and can provide only 2D manipulations [176]. A previously studied modified AFM nanoindentation technique by Ahmad et al. measured the force by using an AFM cantilever and a soft nanoneedle coated tip with bio-adhesive protein [177]. The tip of the cantilever glued to the cell’s surface when moved away from the cell, and the cell elongated and released from the substrate eventually. However, it became difficult to separate the cell adhesion and stiffness from the elongation and cell release process. Additionally, the AFM cantilever used in nanoindentation for cell stiffness characteristics moves in the direction towards the cell. However, the AFM cantilever must move away from the cell in a perpendicular direction to measure the cell adhesion force.

For the implementation of any force measurement system, a high-resolution image and direct observation of system working are needed. The optical microscopy (OM) system is capable of real-time and direct observation of the microscale samples. Still, it has a low-resolution image while dealing with the nano-objects (requires smaller wavelengths than visible light) [178]. However, the AFM system can provide a high-resolution image for nano-objects, but it is unable to provide real-time observation of the sample when the response time is less than a second [179]. Additionally, after AFM scans the cells, they suffer various degrees of injuries, and cell viability depends on it. This may cause remarkable changes in cell morphology [180]. In order to fulfil the lack of the above requirement, environmental scanning electron microscope (E-SEM) was used. Some of the advantages of E-SEM over SEM are that it can observe water-containing samples, and electrically insulating samples do not require metal coating. This gives new opportunities to observe biological samples in their native form directly on the nanoscales range [181].

**Figure 13:**
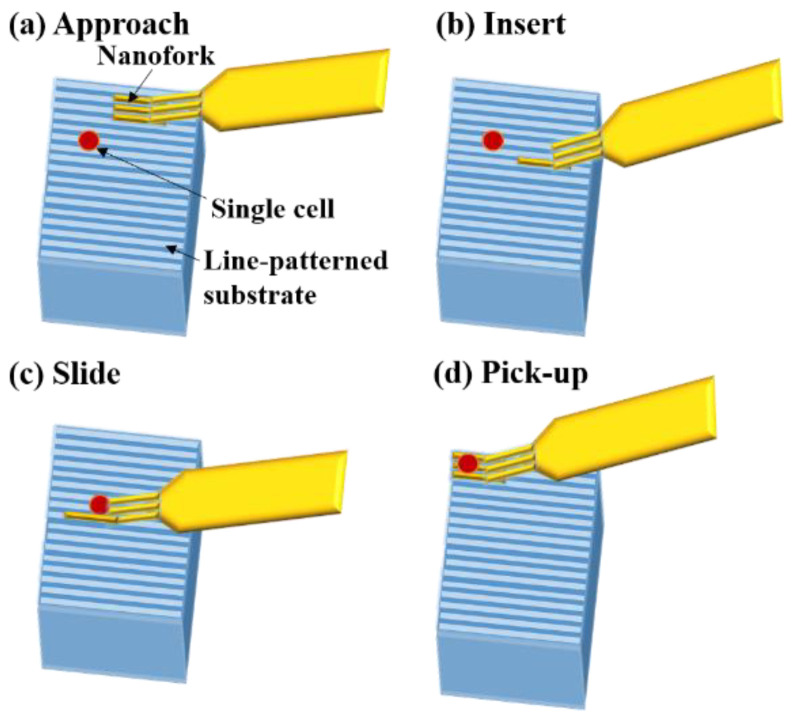
Overview of the: (**a**) cell pick-up and cell adhesion force measurement strategy using nanofork and line array substrate. The line array substrate enables the nanofork to be (**b**) inserted and (**c**) slid between the cell and the base of the substrate and (**d**) to pick up the cell. (Figure redrawn from [182]).

A new strategy was proposed by Ahmad et al. to pick up a whole cell and measure its cell adhesion force simultaneously. A nanofork consisted of three nanoneedles fabricated using an AFM cantilever beam. The cell pick-up and cell adhesion force measurement strategy using a nanofork and a line array substrate is shown in Figure 13a. The line array substrate enables the nanofork to be inserted (Figure 13b) and slid between the cell and the base of the substrate (Figure 13c) and to pick up the cell (Figure 13d). To effectively implement the nanofork, a substrate was designed and structured, with line arrays on the surface of the substrate to be able to provide gaps between the cell and the substrate in order to successfully pick up the cell. A nanorobotics manipulator system inside an E-SEM was constructed for high-resolution images for real-time observation [182]. Shen et al. worked on the single yeast cell adhesion to ITO with various surface energies using a nanorobotic manipulation system inside E-SEM. An end effector (device or tool that is connected to the end of the robot arm which interacts with the cell and environment) was fabricated from an AFM cantilever by focused ion beam (FIB) etching, which was used to detach the single-cell and measure the shear adhesion force [183]. In another study, the AFM cantilever was used to fabricate a micro-puller for the measurement of cell adhesion force and to check the cell viability at the single-cell level. The deformation of the micro puller concluded that the adhesion force of a living cell is much larger than that of a dead cell [184]. A nano-picker was fabricated from an AFM cantilever using a nanofabrication technique. The measurement of the cell-cell adhesion force was based on the deflection of the nano-picker, suggesting that, as the contact-time increased, the adhesion force between the cells also increased for the first few minutes, and then the force became constant [185].

### 5.2. Field-Effect Transistor (FET) Array

The migration of cytotoxic T lymphocytes (CTLs) throughout the body plays a vital role in the defensive immune system by recognizing and eliminating tumorigenic and pathogen-infected cells. CTLs are activated by recognition and interaction between antigen-specific T-cell receptors (TcR) and the major histocompatibility complex (MHC)-I on the antigen-presenting cells (APCs). The immunological synapse (IS) is a tight junction formed where the T cells release cytotoxic granules to the APC cells in high and effective concentration. Additionally, for the activation of the CTLs, the presence and activation of co-receptor CD8 are essential, which helps in specific and prolonged engagement between APCs and T cells and thus the occurrence of actin cytoskeletal and morphological changes to form a stable IS. Lymphocyte function-associated antigen (LFA)-1 is one of the several adhesion molecules which helps to strengthen cell adhesion and enhance IS stability [186]. Thus, it is essential to understand the interactions between these adhesion molecules and T cells at the IS.

Real-time motion tracking microscopy has been able to analyze cell movement, offering the study of migration pattern and polarity in 2D and 3D microenvironments [187]. Moreover, to study and visualize adhesion foci, total internal reflection microscopy (TIRM) was used. However, the expensive instrumentation required additional chemical tags for the identification of the specific proteins involved during adhesion [188]. Microfluidic lab-on-a-chip is an all-electronic sensing approach reported to identify 1000 individual cells per second. It is used to calculate the amount of fluorescence-labelled T cells from a cell mixture [189]. The major limitation of this miniature device is its sensitivity, where the extracellular acidification during T cell activation is detected using label-free electronic readouts in response to the large population of cells [190]. This indicates the signal strength correlating with the cell population. However, T cells function as individuals and are non-adherent cells; a susceptible device for single-cell analysis would be valuable for different applications.

The in-house fabricated field-effect transistor (FET) device demonstrated by Law et al. was mainly used to study T cell migration and adhesion. The transistor surface was coated with different IS formation molecules (such as anti-CD3 Ab, anti-LFA-1 Ab and fibronectin) that would interact with a single T cell further evaluated using impedance spectroscopy. The data of adhesion strength to different antibody and protein coatings were fitted to an electronically equivalent circuit model. Cell-related parameters such as seal resistance (*R*_seal_) formed between the device surface and the cell membrane indicating how tight the cell has adhered on top of the transistor, and combined membrane capacitance (*C*_M_) giving the shape of the cell were obtained [191]. The FET device has also been found useful for the pharmacological platform in cancer studies. In work proposed by Koppenhöfer et al., individual tumor cells (H441, human lung adenocarcinoma epithelial cell line) cultivated on fibronectin-coated FETs were examined for the treatment of silicon nanoparticles [192]. The reaction of cells, when analyzed via microscopic examination, showed that the cells were morphologically changed into round cells, which accompanies the detachment of the cell from the substrate. Thus, the study indicates the application of FETs to analyze the pharmacological effects of new compounds in cancer research.

### 5.3. Quartz Crystal Microbalance with Dissipation (QCM-D) Monitoring

As explained in an earlier part of this review, a complete understanding of bacterial attachment and biofilm formation is necessary for helping to control biofilm development. Various approaches such as the disruption of biofilm with staining assays or colony-forming unit (CFU) counts, molecular expression assays, low load compression tests, and CLSM have provided a great deal of information, including the change in the biofilm development such as biofilm biomass, rheological properties, morphology and gene or protein expression. However, none of the methods are unable to provide insight into the bacteria-surface attachment mechanism. Optical tweezers, flow-displacement devices and AFM methods are used to study the cell adhesion detachment force from the surfaces. However, these methods are unable to extrapolate the information at the microbial community level within a biofilm structure [193]. The microbes organize the 3D structures to communicate within the biofilm, which can be explained by complicated processes such as metabolic changes, genetic clues, and morphological changes. Apart from the destructive micromanipulation technique, such as scrapping, there are limited forces known to remove the biofilm [193].

**Figure 14:**
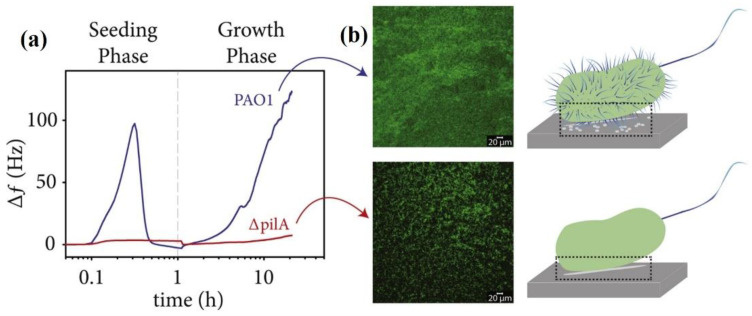
QCM-D for bacterial attachment and subsequent growth. (**a**) Representative requency shifts (Δ*f*) of the 3rd overtone during an initial seeding period and the subsequent biofilm growth period for wild-type *P. aeruginosa* PAO1 and the ΔpilA mutant. The arrows indicate the characteristic recurrent fluctuation in quartz crystal microbalance with dissipation (QCM-D) signal observed between 4 and 6 h *P. aeruginosa* PAO1 biofilm growth. (**b**) Representative maximum intensity projections of CLSM image stacks of *P. aeruginosa* PAO1 and ΔpilA mutant biofilm development on QCM-D crystals. (Reproduced with permission from [195]).

Quartz crystal microbalance with dissipation (QCM-D) can provide non-destructive and non-disruptive information about the substrate-biofilm interface. It senses the mass attached to the oscillatory quartz sensor by a shift in the resonance frequency. As used in the “microbalance”, the negative frequency shift is related to an area-averaged mass via Sauerbrey relation [194] with a sensitivity of ng/cm^2^. The authors employed QCM with dissipation monitoring to study bacterial attachment and subsequent growth, as well as the development of biofilm with the aim to determine the role of pili in the biofilm for surface attachment using wild-type *P. aeruginosa* (PAO1) and pili-deficient ∆pilA mutant PAO1 strains (Figure 14). The study suggested that the pili are an essential factor for coupling the developing biomass to the sensor surface, indicating a dynamic attachment event during the seeding period where the pili cell surface appendages pull the wild PAO1 closer to the surface, thus improving our current understanding of biofilm formation phenomena [195].

### 5.4. Molecular Tension Fluorescence Microscopy (MTFM)

The biological processes from transcription to translation have been dependent on mechanical forces. Many signaling molecules at the cell surface bind to their ligands on adjacent cells or the ECM to mediate mechanotransduction. This ligation bond transmits pN mechanical forces that are generated by the cytoskeleton. Thus, the cryptic sites within mechanosensitive proteins are exposed, which help to modulate the binding kinetics to further fine-tune the mechanotransduction and the corresponding cell behavior. Over the past three decades, cell receptor forces have been measured using two methods. First is a bottom-up approach, the molecular force sensors (MFSs)/TFM (passively detect forces applied onto a substrate) techniques. However, they are widely used by the community of biomechanics research, and the method is sensitive to nN level forces, yielding force sensitivity that is an order of magnitude coarser than the forces applied by individual receptors. In comparison, the second is top-down approaches, which are SCFS/SMFS (actively perturb the cell by external forces) techniques consisting of AFM, OT and BFP. These techniques can measure forces from 1 pN to several nN, but these methods have low throughput and analyze only one bond at a time. Moreover, they fail to capture the whole-cell mechanics or complete activated surface receptors that require clustering in physiological conditions [196].

**Figure 15:**
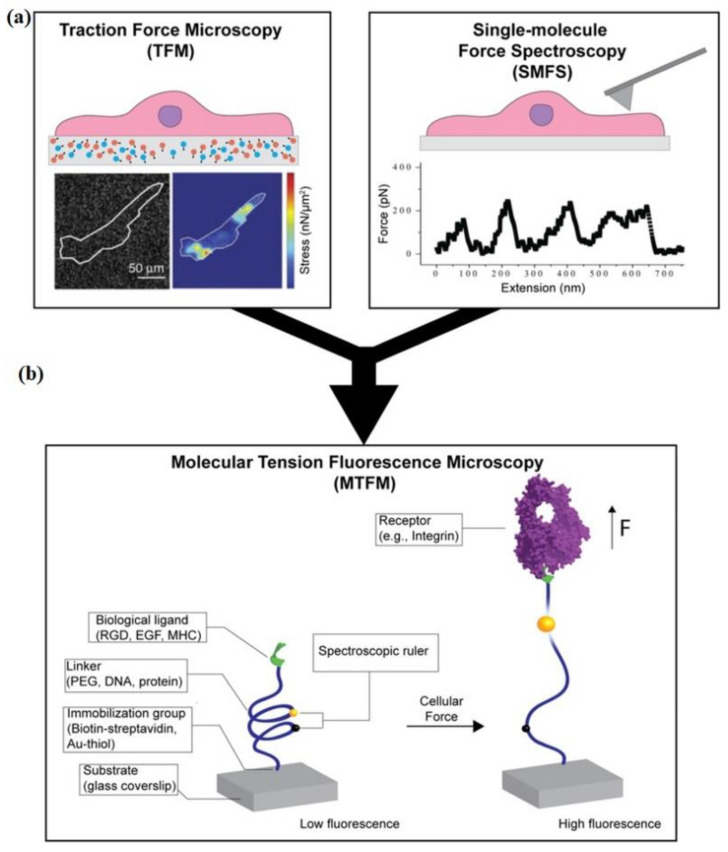
Schematic of current technologies to image cell forces. (**a**) Scheme depicting traction force microscopy (TFM) and single-molecule force spectroscopy (SMFS). (**b**) Simplified diagram that shows how tension probes report on cell forces. (RGD–Arginine-glycine-aspartic; EGF–Epidermal growth factor; MHC–Major histocompatibility complex) (Reproduced with permission from [198]).

Molecular tension fluorescence microscopy (MTFM/MFSs) was developed and consisted of a fluorophore-quencher pair probe separated by a molecular spring immobilized onto the surface. Fluorescence microscopy helps us to visualize probe extension events across the entire cell surface, thus combining the pN sensitivity of SMFS with a high throughput of TFM (Figure 15). Unlike the cross-linked substrates of TFM, the MTFM probe consists of one elastic molecule independently reporting the mechanical forces generated by the bound receptor. The advantages of this strategy are a mapping of tension applied by cell receptors with molecular specificity, millisecond temporal resolution, sub-micrometer spatial resolution, and pN force sensitivity [197] (Stabley et al., 2012). Several recent showcases show the biological applications using this MTFM probe [196,198].

### 5.5. Variable-Angle Total Internal Reflection Fluorescence Microscopy (va-TIRFM)

The early stage of the fundamental cellular processes (such as proliferation, migration and mechanotransduction) takes place at focal adhesion contact that involves plasma membrane receptors (e.g., integrins) which recognize ligands in the environment known to be the specific binding. Apart from that, a broad range of nonspecific interactions such as polymer steric repulsion forces, electrostatic forces, van der Waals forces or undulation *Helfrich* forces play a vital role in the cell, interacting with their microenvironment [199]. These forces are distance-dependent and thus can be analyzed by measuring the distance between substrate and plasma membrane. Many studies have shown that the nonspecific and specific forces tend to have a cooperative action, e.g., integrin-mediated signaling and adhesion is impacted by the glycocalyx (known to exert nonspecific steric repulsive forces) [200]. Moreover, according to Paszek et al., it is presumed that in tumor cells, integrin spatial organization and function is mechanically enhanced by the glycocalyx [17]. Recently, various approaches have been studied to explore cell-microenvironment interactions such as PDMS micropillar array to measure cell-generated forces, microfluidic devices or fluid microjets to analyze cell adhesion using shear forces, AFM or OT to study local measurements on plasma membrane proteins, standard TIRF microscopy and interferometric photoactivated localization microscopy (iPALM) to observe specific adhesion. These techniques are beneficial to decode complex processes such as mechanotransduction, but not nonspecific interactions.

**Figure 16:**
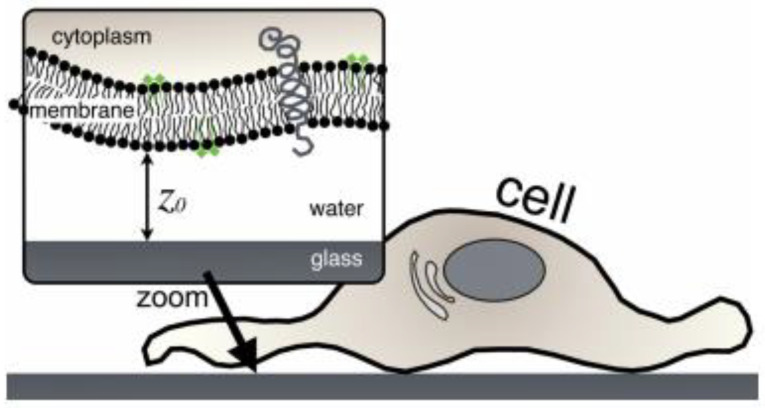
Schematic drawing of a cell spread on a glass substrate. Inset: the plasma membrane is labelled with DiO (green, fluorescent, lipophilic carbocyanine dye). z_0_ is the distance from the substrate to the plasma membrane. (Reproduced with permission from [202] under Creative Commons Attribution 4.0 International License).

The new method to quantify single-cell adhesion strength is based on variable-angle total internal reflection fluorescence microscopy (va-TIRFM) [201]. The technique involves a gradual increase in the incident angle of the light beam on the sample while recording a stack of multiple TIRF images with an acquisition rate of about one second. Further, these processed images can be useful in restoring the cell topography with a nanometric axial resolution of typically 10–20 nm, i.e., a map of distance z_0_ between the substrate and the stained membrane, as shown in Figure 16. The present technique is not based on single-molecule detection. Consequently, the observation sampling rate is at ≈1 s with a surface density of energy of about ≈ 10–20 J/cm^2^. This energy is significantly smaller than the energy in super-resolution techniques such as PALM or Stochastic Optical Reconstruction Microscopy (STORM) (10^2^–10^3^ kJ/cm^2^) and stimulated emission depletion (STED) microscopy (10^4^ kJ/cm^2^). Thus, this irradiation lethal energy dose used by these super-resolution microscopic techniques leads to the photodamage of the cells during the exposure time, while va-TIRFM does not induce photodamage to the specimen. Furthermore, specific adhesion zones can be observed using an amphiphilic dye molecule to label the plasma membrane, and this helps to measure the distance between membrane-substrate. Thus, minimum cell preparation with no particular adhesion protein labeling is required. Additionally, the cells do not need to be fixed. Therefore, real-time nondestructive observations with dynamic aspects of the adhesion process are possible, where the cell migrates freely over the substrate. Finally, the axial measurement using va-TIRFM is ~300–400 nm as compared to the limited range (100–150 nm) of fluorescent techniques previously cited [201]. Thus, this phenomenon is crucial to measure the nonspecific interactions which induce high forces that repel the cell far from the substrate (>100 nm). To conclude, va-TIRFM can be implemented on any TIRF microscope due to the fine control setup of the light beam incident angle [202].

### 5.6. Non-Contact/Non-Destructive Approach

Numerous studies have reported the existence of a strong correlation between biological cell elasticity and adhesion properties’ irregularity and the progression/pathogenesis of various abnormalities. For example, due to the lower adhesion and elastic stiffness of metastatic invasive tumor cells, they detach from the primary tumor site and reach the secondary site via blood/lymph vessels [203]. Thus, the determination of the stiffness and adhesion properties of the cell will indicate its biological state (e.g., malignant or benign). Various experimental techniques reported in the last few decades, such as AFM, micropipette aspiration, cell poking/cell compression, E-SEM, osmotic swelling/shrinking and micromanipulation techniques, require direct physical contact with the cell sample. This causes inadvertent effects such as altering its shape and triggering a mechanotransduction mechanism that may directly affect the validity and accuracy of the measurement.

**Figure 17:**
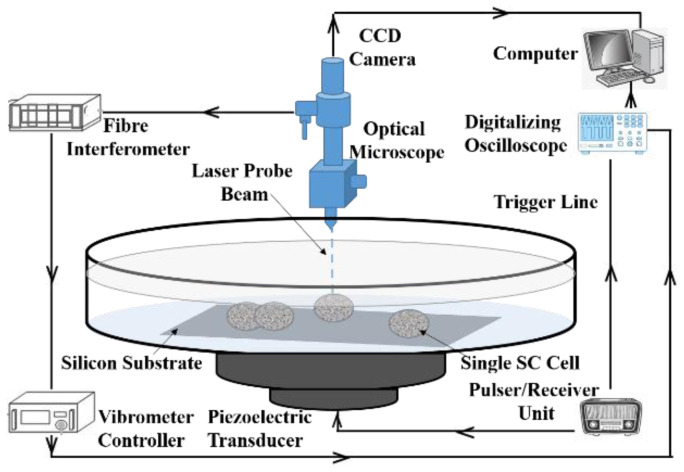
The instrumentation and connectivity diagram of the experimental set-up (not to scale) (CCD–Charge coupled device; SC–single cell) (Figure redrawn from [204]).

The work by Farzi and Cetinkaya demonstrated a non-contact/non-destructive approach to acquire adhesion and mechanical properties of single *Saccharomyces cerevisiae* (SC) cells (baker’s yeast) based on interferometric (optical) motion detection and ultrasonic excitation [204]. The set-up developed for the present experimental study is shown Figure 17. With the help of this current approach, the rocking (rigid body) and the internal vibrational resonance frequency of the single SC cell can be acquired. Thus, related by mathematical models to their surface and mechanical properties such as Young’s modulus, the surface adhesion energy and surface tension of the cell membrane as compared to the optical stretching technique, where intense laser heating influences the enzymatic activity of the cells and the viscosity of the medium, cause convection currents that may result adversely in accuracy measurement [205]. The present approach uses acoustic waves that cause weak deformations at nm scale-level with a lower power laser beam, thus leading to the reduced alterations in their physical properties and the risk of damaging cells thermally.

### 5.7. Inverted Pulsed Opto-Acoustic Microscope (iPOM)

Many fundamental processes, such as the progression of degenerative diseases, morphogenesis, mechano-transduction and motility, recognize cell adhesion strength and stiffness as their key players. Thus, to analyze the cell response in in vitro conditions, artificial substrates need to mimic bioimplants or the extracellular environment [206]. Cells attach to these surfaces at a distinct area known as focal adhesions, where the substrate and cytoskeleton anchor together, forming mechanical forces. This leads to the large scale organization of the cytoskeleton, which therefore impacts the stiffness of the whole cell. To understand the stiffness completely, it is necessary to probe the stiffness. However, the existing technologies that can study at the subcellular scale can only provide images of cell adhesion. The adhesion patches are observed as accumulated adhesion proteins (e.g., vinculin) with the help of fluorescence microscopy. However, the areas of close contact, which are an integral part of the cell-substrate interface (not limited to focal adhesion), are observed with spatial distribution, and it does not relate to the presence of vinculin. The TFM thus helps to monitor such passive contacts where the motion of fluorescent beads inserted in the substrate is tracked [207]. However, the cell deforms the substrate passively in some parts while deforming it actively in the regions near the adhesive patches. Thus, to gain complete insight into cell-substrate interactions, innovative imaging technology is needed, where separate passive and active adhesion patterns could be monitored simultaneously. Moreover, the existing modalities use contact probes at a sub-cell resolution such as AFM, nanostructured substrates with a 2D array of contact probes or pressurized nanopipettes, which may interfere with the normal functionality of the cell [208].

**Figure 18:**
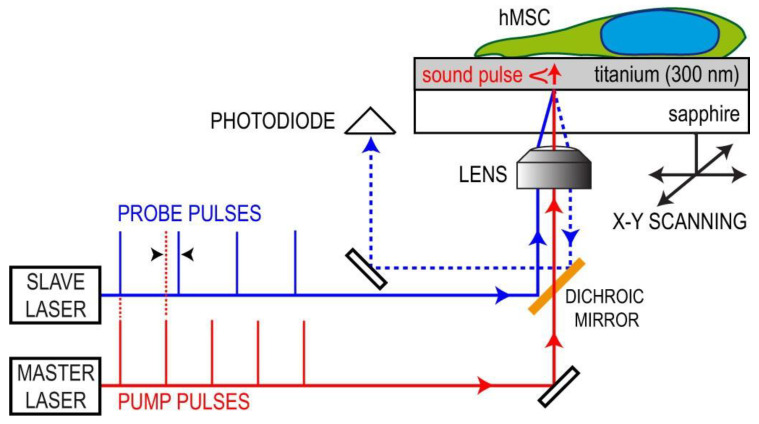
Side-view of the inverted pulsed opto-acoustic microscope (iPOM). Two lasers are used with a slightly different repetition rate to generate broadband acoustic waves and detect echoes in the Ti transducer by optical sampling. (hMSC—human mesenchymal stem cells) (Reproduced with permission from [209] under Creative Commons Attribution 4.0 International License).

Laser-generated GHz-ultrasonic based technology can image single-cell adhesion and stiffness measurements simultaneously. In particular, an inverted pulsed opto-acoustic microscope (iPOM) that operated in the range of 10–100 GHz has been recently demonstrated to be well suited for the remote study of cell adhesion with high-resolution images [209]. The mechanical properties of the cell and the rigidity of the interface at the nanoscale can be analyzed using the frequency-dependence of the acoustic reflection coefficient. Abi Ghanem et al. cultured human microvascular endothelial cells (hMEC-1) and human mesenchymal stem cells (hMSCs) on a rigid substrate at different culture times to analyze isolated interacting cells and the impact of cell-cell contacts [210]. They used an iPOM to generate acoustic pulses up to 100 GHz frequency in the substrate and to monitor different parameters for various cell lines, to reveal passive and active adhesive processes at the single-cell level. The schematics of the experimental set-up of iPOM are given in Figure 18. They concluded that the active adhesion process could be quantified with a ratio of the real to nominal contact areas, while the mean interfacial stiffness helps to quantify passive adhesive processes. Such analysis helps to separate the two different adhesion processes and observe the correlation simultaneously with the mechanical reorganization of the cell, which can be an essential application in tissue engineering.

### 5.8. Traction Force Optical Coherence Microscopy (TF-OCM)

The field of mechanobiology seeks a particular interest in cellular traction forces (CTFs) to understand the role of mechanical forces and interactions in metastasis, collective cell migration and angiogenesis. Thus, TFM was developed to measure the forces exerted by the cells as they interact and sense their environment based upon the optical measurement of CTF-induced deformations. TFM helped to investigate various biological findings, such as the connection between metastatic cancer cells and strong CTF generation [107], and that the neurons from central nervous system (CNS) exert stronger force than that of the growth cones of neurons from peripheral nervous system (PNS) [211]. With growing demand, the technical challenges and complications in the imaging system of the TFM to study the mechanical behavior of a single-cell in the 3D environment [212], a wide range of spatiotemporal scales [213] and CTFs within biopolymer substrates [214], there was a need for high-resolution imaging to view large fields, which could be achieved with the help of high temporal sampling and repetition over an extended duration. This was all possible with 3D TFM using confocal fluorescence microscopy. However, the range of experimental conditions for 3D TFM was restricted, which included a limited penetration depth of a few hundred micrometers in scattering media, complications caused by phototoxicity and photobleaching, and the long measurement time required to acquire larger volumes.

Due to these limitations of TFM with the current technologies, Mulligan et al. proposed TFM based optical coherence microscopy (OCM) [213]. This is a modification of optical coherent tomography (OCT) along with the high transverse resolution. Thus, the method was named traction force optical coherence microscopy (TF-OCM). It offered several advantages consisting of the combination of computational imaging methods and OCM. The benefits of TF-OCM included enabling the quantitative reconstruction of 3D CTFs, high temporal sampling in scattering media, volumetric acquisition rate in minute-scale with the help of a Fourier domain OCM system and scattering. Phototoxicity and photobleaching concerns were reduced with the use of label-free imaging at near-IR wavelength, and computational adaptive optics (CAO) helped to achieve focal plane resolution at extended depth-of-field. Moreover, Mulligan et al. demonstrated the feasibility of TF-OCM, where they reconstructed volumetric OCM data with CAO procedures to measure time-lapse deformations caused by the extracellular matrix resulting from CTFs in 3D hydrogel substrate. The matrix deformations revealed the difference of dynamic force exerted between normal and contractility-inhibited NIH-3T3 fibroblasts [213]. They also expanded the above method and used this new technique to quantify the time-varying 3D CTFs exerted by NIH-3T3 fibroblasts embedded in Matrigel substrate [215].

### 5.9. Upconverting Spinners (UCSPNs)

Nowadays, massive approaches have been used for cell or bacteria detection in bio-fluids that are unable to detect *single-cells* or bacteria. The traditional bacterial detection approaches are based on increasing the population size of the bacteria in the sample to reach a specific detection level, which in return requires more than 24 h. This is only possible if the initial seed sample of the bacteria is more significant than the threshold value. Moreover, for reliable outputs, the conventional hematology analyzer requires a large number of cells to detect anomalous cancer cells in blood samples. Thus, the present challenge is to develop sensitive, accurate, yet rapid methods, which are capable of detecting both cells and bacteria simultaneously at the single-body level. To achieve this, various immunoassay techniques have been developed with bioconjugated nanoparticles, assays with kinetic exclusion and on-chip microfluidic platforms, which requires sophisticated instrumentation making it expensive, complex sample manipulation and considerable response time. Thus, bio-photonic and optical methods have emerged with a possible alternative.

**Figure 19:**
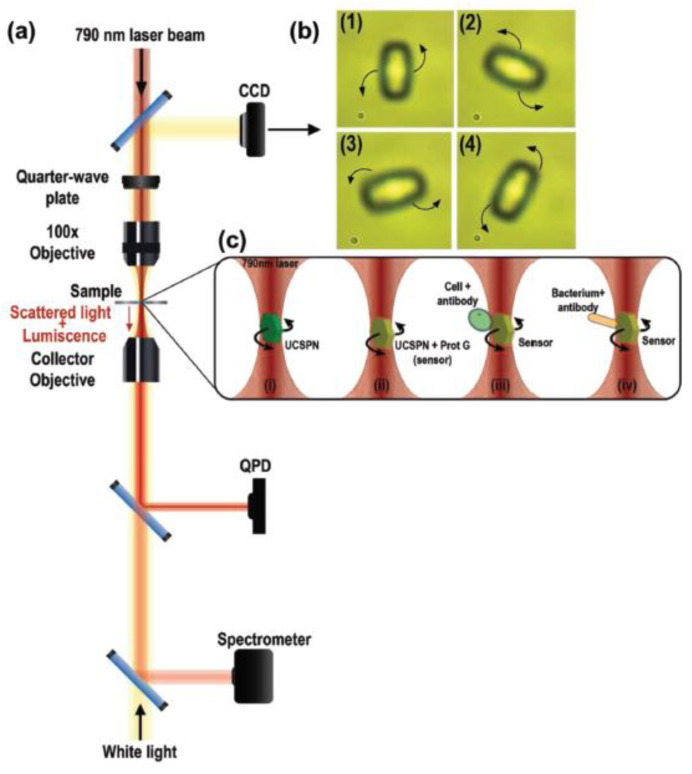
Upconverting spinners. (**a**) Schematic representation of the experimental set-up for optical trapping and rotation of Upconverting spinners (UCSPNs) and single-body biodetection. (**b**) The rotation speed of the UCSPN is determined by recording the intensity fluctuations of the transmitted laser beam using a Quadrant PhotoDetector (QPD) or polarized emission using a spectrometer and analyzing them in the frequency domain. Real-time visualization of the UCSPN, bacteria, and cells are possible, and some representative pictures of a UCSPN are included. (**c**) The different situations evaluated all along this work are also schematically represented: (**i**) trapping and rotation of a single and bare UCSPN, (**ii**) surface-functionalized UCSPN, (**iii**) surface-functionalized UCSPN with an attached cell, (**iv**) surface-functionalized UCSPN with an attached bacterium. The attachment of either a bacterium or a cell to the optically rotating UCNP leads to a remarkable change in the rotational velocity that is quantified from the frequency spectrum. (Reproduced with permission from [218]).

The Upconverting particles (UCPs) used in bio-photonics are efficient for infrared-to-visible optical conversion via the absorption of infrared photons. This helps in the accession of high resolution and low background bioimages. They have been extensively used for intracellular biosensing due to their background-free, bright and temperature-dependent luminescence [216]. At the same time, optical trapping is a contactless technique used for single-cell and bacteria studies, with accurate rotational and translation control over micro and nanostructures. According to Galajda and Ormos, the single-laser beam can trap as well as induce the rotation of birefringent UCPs [217]. Thus, the rotational dynamics of these Upconverting spinners (UCSPNs) have been introduced as a potential high-sensitivity sensor. The schematic representation of the optical trapping and rotation of UCSPNs and single-body biodetection is shown in Figure 19. In work described by Ortiz-Rivero et al., UCSPNs have been used for the accurate and contactless detection of single bacteria and pathogenic yeast cell attachment events. This was achieved by the real-time monitoring of the rotational velocity of hexagonal erbium and ytterbium codoped NaYF_4_ microparticle. The cell adhesion of a single bacteria is evidenced by an angular deceleration using a biomolecule functionalized surface and real-time monitoring of visible luminescence or transmitted laser beams by UCSPNs. Thus, UCPs demonstrate their potential for the development of high-sensitivity, fast and cost-effective systems for the detection of single-cells [218].

### 5.10. Fluidic Force Microscope (FluidFM)

In biological science, single-cell adhesion (SCA) force plays a vital role. However, the present techniques fail to have in-detail investigation due to their low throughput and the lack of temporal resolution. Since the SCFS method helps to monitor and manipulate one cell at a time, it can hardly be used for the accountability of single-cell variability. The AFM based methods have limited throughput as it measures few cells per day and does not provide the kinetic evaluation for the cell adhesion force over a longer time-scale. Strohmeyer et al. demonstrated the adhesion kinetics between live-cell and substrate by varying the time of contact [219]. As it is not recommended to detach a fully adhered and spread out cell, thus the method can only be beneficial for molecular level investigations. Moreover, separate cantilevers are required to be calibrated and functionalized for every force curve measurement, thus making the process slow and able to measure the detachment event of a single-cell in a matter of hours. Since the adhesion process can be examined at only a particular instance, the adhesion kinetics cannot be studied. Furthermore, the cantilever of AFM is fixed on the top of the cell, which is an additional issue, as unwanted elements are added to the microenvironment of the desired study.

The fluidic force microscope (FluidFM) is the nanofluidic extension of AFM [220]. The AFM cantilever is introduced with the nanofluidic channel attached to a refillable fluid reservoir, regulated by a pressure control system. The FluidFM micropipette cantilever has a unique and non-standard structure as compared to AFM. The users can dispense or collect femtoliter scale fluids with this hollow cantilever microfabrication technology. The FluidFM technology has been used for injection or extraction of liquids into or from the living cells [221], combining SCFS [222] and the colloidal probe technique [223]. It also has excellent potential in obtaining large quantities of data and yielding statistically relevant biological information. Dörig et al. demonstrated the detachment of neural cells from a glass slide functionalized with fibronectin to measure the adhesion forces using a FluidFM micropipette cantilever [222]. Potthoff et al. used FluidFM technology to demonstrate the quantitative bacterial adhesion measurements of cell-cell and cell-substrate interaction that are applicable in biofilms and infection biology by showing the detachment forces for *E. coli* and *S. pyogenes* bacteria strains from polydopamine treated surfaces [224]. Jaatinen et al. presented the effect of external electric current on the mechanical properties and cell adhesion of the C2C12 mouse myoblast cell line [225]. The FluidFM technology provided a serial quantification and detailed analysis of cell adhesion and its dynamics. Sankaran et al. performed SCFS to measure cell adhesive strengths on different surfaces with noncovalently immobilized peptide ligands [226]. They proved using FluidFM that cells adhere to surfaces with RGD in a regular covalent manner and a dynamic non-covalent manner. Sancho et al. introduced a methodology using FluidFM to evaluate cell-cell adhesion forces with mature intracellular contiguity in the cell monolayer [227]. The detachment studies showed that the L929 fibroblasts (on glass substrate) exhibited negligible cell-cell adhesion, while the human umbilical artery endothelial cells (gelatin-coated glass) exerted strong intracellular adhesion forces per cell.

**Figure 20:**
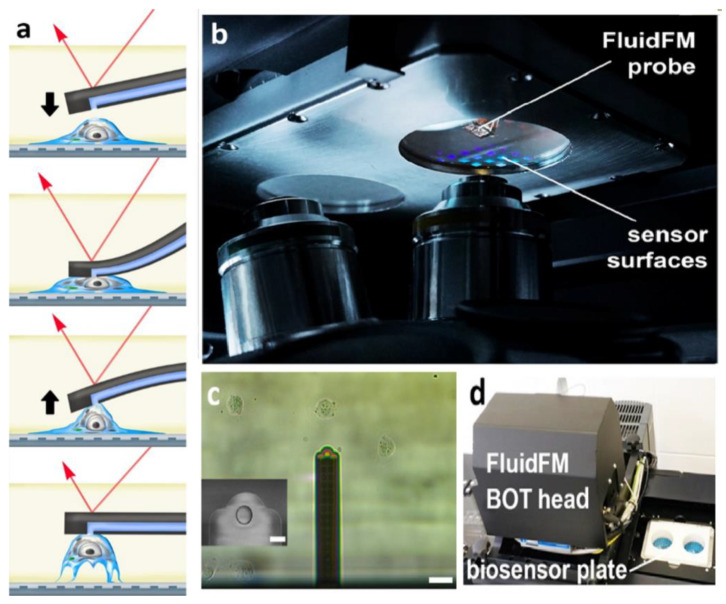
Principle of the robotic fluidic force microscopy (FluidFM BOT) measurements. (**a**) The workflow of the cell adhesion strength measurement using the FluidFM method. The hollow cantilever is approached the adhered cell (top frame). Upon contact, a vacuum is applied to fix the top of the cell to the cantilever (middle frame). Once a stable connection is established, the cantilever is retracted while its bending is recorded by the reflected laser beam deflection (red arrow). (**b**) The custom-made biosensor insert holder positioned in the sample holder of the FluidFM BOT device. The photograph is made from below, showing the objectives of the inverted microscope of the FluidFM BOT, the two circular wells, the sensor surfaces (reflective coloured squares in the right well) and the probe on the upper side of the sensor insert. (**c**) Live view image of the fluidic force microscopy measurement: the picture shows the cantilever (and the purple laser spot on it) with surrounding adhered cells (scale bar: 40 μm). Inset: scanning electron microscopy image of the top of the cantilever. The aperture with an 8 µm diameter can be seen. Scale bar: 8 μm (Image provided by Cytosurge AG). (**d**) Photograph showing the FluidFM BOT device on an anti-vibration table. The stage is fixed on an inverted microscope with the head unit above the objective. The inserted biosensor plate is seen at the right. (Reproduced with permission from [24] under Creative Commons Attribution 4.0 International License).

In the study by Sztilkovics et al., a high spatial and temporal resolution resonant waveguide grating based label-free optical biosensor was combined with robotic fluidic force microscopy (FluidFM BOT) to monitor and record real-time adhesion force kinetics of more than 300 HeLa cells over 1.5 h. The schematic and principle of the FluidFM BOT are shown in Figure 20. The robotic device was used here, and it can study single-cells over mm-cm scale areas as compared to the traditional FluidFM, where the manipulation range was only about 300–400 micrometers. This measurement feature has remarkably increased the throughput and employed the use of a microplate-based large area biosensor. The biosensor signals, along with the direct force measuring technology, were calibrated and focused on 30 individual cells. This method helped to separate adhered cells mechanically and directly from the surface of the biosensor and evaluate the cell adhesion kinetics, which can be assessed for extracting maximum adhesion force and energy values of a large cell population. Here, Hela cells showed a higher level of heterogeneity based on the adhesion process explained by three parameters, such as saturation value for the adhesion force, a maximum speed of the adhesion process and final cellular area. Such a single-cell adhesion experimental database will help us to understand the heterogeneity of single-cell phenotypes [24].

In another study by Saftics et al., the micropatterning of living mammalian cells on carboxymethyl dextran (CMD) hydrogel layers using the FluidFM BOT technology has been presented [228]. CMD films are generally used in label-free biosensor applications. Here, CMD layers with thicknesses of several tens of nanometers provided support for the controlled adhesion of living cells. Cell micropatterning on the CMD surface was obtained by printing cell adhesion mediating cRGDfK peptide molecules (cyclo(Arg-Gly-Asp-D-Phe-Lys)) directly from an aqueous solution using a microchannel cantilever with the subsequent incubation of the printed surfaces in the living cell culture. Cell patterns with different geometries (spot, line, and grid arrays) covering both micrometer and millimeter-centimeter scale areas were shown. The adhered patterns were analyzed by phase-contrast microscopy, and the adhesion process on the patterns was real-time monitored by digital holographic microscopy, enabling the quantification of the survival and migration of cells on the printed cRGDfK arrays. Table 2 summarizes the advantages and limitations of new techniques used to study single-cell adhesion.

### 5.11. Resonant Waveguide Gratings (RWG)

Under in vivo conditions, several components are involved in cell adhesion, and they interact in a complicated and tightly controlled manner. These components are carbohydrates and proteins of the ECM, cell adhesion receptors and other soluble factors (such as ions and small molecules) regulating the interaction. Due to the experimental difficulties, the quantitative data of cellular adhesion obtained through most of the experimental models can be considered as a strong simplification of the in vivo situation. However, most of the experimental methods available to measure cell adhesion and cell-surface interaction have serious disadvantages when a multi-component model of cell adhesion has to be quantitatively investigated in a time frame.

A label-free biosensor is a common tool which does not require the application of fluorescent dyes and is mainly used for the measurement of cell adhesion, proliferation, spreading, differentiation, migration, signal transduction analysis, receptor-ligand binding and cytotoxicity. These techniques are useful to investigate cell-surface interaction kinetics. However, detection capacity and sensitivity are found to be low in label-free detection. Recent developments have overcome these limitations, which shows high throughput capability for the practically parallel measurement of hundreds of samples in microplate format. The required sensitivity of being able to detect the binding of ligands of molecular mass as low as 100–200 Da, below 5 pg/mm^2^ surface mass density, has been easily met, and their current throughput allows up to 460,000 data points/h. These techniques include electric cell-substrate impedance sensing (ECIS) [229], photonic crystal-based sensors [230] and resonance waveguide grating (RWG) [231].

Peter et al. studied high-throughput resonant waveguide grating multicomponent model systems of cell–surface interactions [232]. The interaction of the anti-adhesive, antifouling coating, PLL-g-PEG and its RGD (Arg-Gly-Asp) functionalized form, PLL-g-PEG-RGD, with the green tea polyphenol EGCg was monitored in situ. Right after, cellular adhesion on the EGCg exposed coatings was recorded in real-time. Despite the reported excellent antifouling properties of the above polymer coatings, EGCg strongly interacted with them and affected their cell adhesivity in a concentration-dependent manner. Moreover, it has been proven that optical waveguide-based sensors are capable of investigating not just biological samples but also nanoparticles and self-assembled nanostructured coatings as well [233,234]. RWG has been applied to investigate cell signaling mediated through the epidermal growth factor (EGF) receptor [235], cytoskeleton modulation [236], and the G-protein coupled receptor (GPCR) bradykinin B_2_ receptor [237,238].

## 6. Limitations and Future Prospects

**Figure 21:**
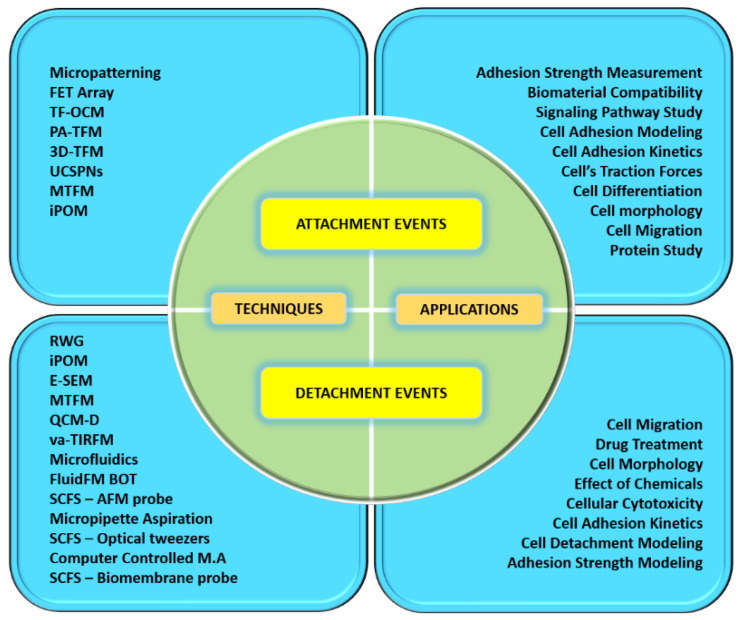
Summary of the techniques involved in single-cell adhesion studies categorized by the adhesion attachment and detachment events and their applications.

Nowadays, single-cell and single-molecule research interest is increasing day by day for various biological and biomedical applications. In the last couple of decades, different techniques have been developed to probe the cell generated forces. The manipulation techniques have provided an effective approach for single-cell analysis, through which cells can move, divide, differentiate, flow, sense, and remodel in their microenvironment. These physical forces (forces that are developed from within the cell via the cytoskeleton, i.e., endogenous forces, or come from outside the cell, i.e., applied forces) are not just the simple switches of mechanotransduction, but a mechanism to communicate within and between cells. Figure 21 summarizes the techniques involved in single-cell adhesion studies categorized by the adhesion attachment and detachment events and their applications. Even though the various techniques up-to-date have achieved many successes, there are still many challenges that need to be addressed for single-cell analysis.

In this review, we have overviewed current technologies used to quantify cell-generated forces. However, these techniques are restricted for in vitro systems and may be affected due to the low signal-to-noise ratio. The environment around the molecular interactions, which involves force sensors of the instrument, does not reflect that present in real cellular interactions. This is because of the solid surfaces, which are used for cell adhesion and do not accurately provide the lipid bilayer membrane fluidity or the viscoelastic nature of the cell membrane and the cytoskeleton. The conjugation of the sensor to the biological receptors can affect the diffusion rate of the molecule, which may alter the binding kinetics.

The current sensors used for force measurement constructed methods are limited to the type of receptor-ligand interactions that can probe. The attachment of membrane proteins remains challenging, which limits the adhesion interactions of soluble proteins as ligands. Thus, a better characterization technique is needed in terms of their kinetic effects on the biological receptor-ligand conjugates. This is crucial as the recent data have shown that the instrument tends to influence the molecules which are conjugated.

In most of the cell adhesion force sensor studies, cell-substrate interactions are mainly focused upon due to the simple sample preparations and imaging conditions. It becomes challenging to study the molecular forces applied by the cell on substrates as well as on the other cell membranes, i.e., cell-substrate and cell-cell interactions in 3D, due to the difficulties faced such as sensor degradation, bioconjugation and imaging. Though many recent developments have been proposed using 3D analysis, it is significantly challenging to study due to the challenges involved in inserting an exogenous probe into an established interface. In addition, many interactions consist of two-way processes, where the probes need to be attached or endocytosed through the cell membrane while waiting to interact with the other molecules.

In the future, SMFS on proteins could play an essential role in the development of therapeutic proteins. These therapeutic molecules must be colloidally (should not aggregate or denature) and biophysically (during manufacturing, storage, shipping or administration) stable, as target binding and achieving their biological goal of signaling pathway alone does not makes the molecule a viable drug. Thus, SMFS techniques can help the pharmaceutical industries to be able to screen molecules where early-stage detection will help to determine their biophysical stability.

Before opting for one technique over others, an investigator needs to consider various factors besides the sensor nature and the mechanical assumptions associated with it, such as whether the samples are in in vivo or in vitro, physically accessible for contact or not, and in 2D or 3D; whether the dynamic process requires time-lapse measurement or one-time point quantification; nanoscale vs. microscale; force resolution (pN to nN), etc.

Despite all the challenges faced, the technology gives us deep insights into the mechanisms involved in cellular processes. All these current instruments were born from the field of physics and engineering. Still, researchers need to build their customized setups, which would help in high-potential elucidations of mechanisms in biology. In addition to the instrumentations, there should be considerations of theoretical algorithms to analyze the datasets to extract maximum information from them.

## 7. Conclusions

In the biological system, cell adhesion is a fundamental process. Both cell attachment and detachment studies provide crucial knowledge regarding many vital processes, leading us to discover the causes that may trigger various diseases and hence help us develop a strategy to cure them. Numerous adhesion assays have been developed for cell adhesion studies, which are applicable in a wide range of fields. Every advanced method is unique for its specific importance and independent of its purposes, making them difficult to compare. For cell adhesion studies, finding the appropriate method that is applicable is dependent on the purpose of the information being obtained. Thus, single-cell research is essential to fully understand the behavior of a cell and its function in the human body.

## Figures and Tables

**Table 1:** Comparison of advantages and limitations in the techniques used for cell adhesion studies.

Methods	Strength	Weaknesses	References
Polyacrylamide—Traction Force Microscopy (PA-TFM)	Real-time observation; Mechanical and chemical adjustment flexibility; No expensive equipment needed; Fabrication is inexpensive; Adaptable to a variety of cells	Needs to record both unstressed and stressed state of the substrate; Suffers from uncertainties in tracking beads’ position	[107,108]
Three Dimensional Traction Force Quantification (3D-TFM)	Real-time observation; Flexible to chemical and mechanical adjustment; Adaptable to a large variety of cells; Flexible to chemical and mechanical adjustment; Adaptable to a large variety of cells	It needs a high-end confocal microscope; Requires high computational processing; Suffers from uncertainties in tracking beads’ position; Limited penetration depth in scattering media; Complications caused by phototoxicity and photobleaching; Long measurement time required to acquire larger volumes	[109,110]
Micropatterning (Micropillar/Micropost)	Real-time observation; Force quantification easier; The micropillar stiffness is manipulated by its geometry; Gives good precision over surface chemical properties on the micrometer scale	The substrate can alter a cell’s behavior; Requires sophisticated equipment to fabricate; Needs a skilled operator; The sensitivity of the microposts to the particular cell type needs to be optimized	[111,112]
Micropipette Aspiration	Real-time observation and measurement; Common lab equipment	Alignment of probe and cell; High skilled (experienced) operator; Operator variable; Cell damage (hard contact)	[113,114]
Computer Controlled Micropipette	Higher sensitivity; Fewer side effects; Measured in a relatively short time period	Alignment of probe and cell requires micromanipulator; Expensive equipment	[62,115]
Atomic Force Microscopy	Real-time observation; Precise data for short term adhesion studies	Alignment of probe and cell requires micromanipulator; Time-consuming; Need a skilled operator; Operator variable; Cell damage (hard contact); Expensive equipment; Not real-time measurement	[116,117]
Biomembrane Force Probe	Real-time observation; Precise data for short term adhesion studies	The low maximum force (pN); Restricted to short term adhesion; High skilled (experienced) operator; Operator variable; Probe variable (fluctuation of probe due to thermal excitation)	[79,118]
Optical Tweezers	Real-time observation; Precise data for short term adhesion studies; Compatible with a microfluidic device	The low maximum force (pN); Restricted to short term adhesion; High skilled (experienced) operator; Operator variable; Cell damage	[119,120]
Microfluidics	Straightforward construction and operation; Real-time observation and measurement; Convenience in size (compatible with cell sizes); Fast and simple to operate; Non-invasive to cell	Low detachment force; Restricted to short-term adhesion	[121,122]

**Table 2:** Comparison of advantages and limitations in the recent techniques used for cell adhesion studies.

Methods	Strength	Weaknesses	References
Nanorobotics manipulator system inside an Environment Scanning Electron Microscopy (E-SEM)	Real-time observation; High-resolution images; Minimum damage to the cell	The cell requires a fabricated micromanipulator; Time-consuming; Needs a skilled operator; Expensive equipment.	[182,183,184,185]
Field-Effect Transistor (FET) Array	No expensive chemical tags required; The system can be applied to migrating cells	Sensitivity relies on the adhesion strength of the individual cell; Performance is mainly limited by the parasitic effect caused by chip design; Requires sophisticated equipment	[191,192]
Quartz Crystal Microbalance with Dissipation (QCM-D) Monitoring	Versatile technique; Real-time characterization of the dynamics at the cell-biomaterial interface; Provides non-destructive and non-disruptive information about the substrate-biofilm interface; The sensitivity of ng/cm^2^	High skilled and experienced operator; Operator variable; Cell damage (hard contact)	[193,195]
Molecular Tension Fluorescence Microscopy (MTFM)	No cross-linked substrates like TFM; Mapping of molecular tension applied by cell receptors with molecular specificity, millisecond temporal resolution, sub-micrometer spatial resolution, and pN force sensitivity.	It needs a high-end confocal microscope; Requires high computational processing; High skilled and experienced operator	[196,197,198]
Variable-Angle Total Internal Reflection Fluorescence Microscopy (va-TIRFM)	No photodamage of cells; Specific adhesion zones can be observed using an amphiphilic dye molecule to label the plasma membrane; Minimum cell preparation required; No particular adhesion protein labeling; Real-time non-destructive observations	Not based on single-molecule detection; Needs high computational processing; A highly skilled and experienced operator	[201,202]
Non-contact/non-destructive approach	Reduced alterations in the physical properties and risk of damaging cells thermally.	Needs high computational processing; High skilled and experienced operator; Validity and accuracy of measurement are essential	[204,205]
Inverted Pulsed Opto-Acoustic Microscope (iPOM)	Well suited for the remote study of the cell adhesion with high-resolution images; Operated in the range of 10–100 GHz	Expensive equipment; Time-consuming; Needs a skilled operator	[209,210]
Traction Force Optical Coherence Microscopy (TF-OCM)	High transverse resolution; Enables quantitative reconstruction of the 3D cellular traction forces (CTFs); High temporal sampling in scattering media; Volumetric acquisition rate in minute-scale with the help of Fourier domain optical coherence microscopy (OCM) system; Phototoxicity and photobleaching concerns were reduced with the help of label-free imaging at near-IR wavelength; Computational adaptive optics (CAO) helped to achieve focal plane resolution at extended depth-of-field	Needs high computational processing; Needs to record both unstressed and stressed state of the substrate; Suffers from uncertainties in tracking beads position; Expensive equipment; Time-consuming; Needs a skilled operator	[213,215]
Upconverting Spinners (UCSPNs)	High resolution; low background bioimages; Intracellular biosensing due to their background-free, bright and temperature-dependent luminescence	Need a skilled operator; Operator variable; Expensive equipment	[216,217,218]
Fluidic Force Microscope (FluidFM)	Record real-time adhesion force kinetics in minimum time scale; Increased throughput	Needs a skilled operator; Needs high computational processing; Operator variable; Expensive equipment;	[24,221,222,223,224]
Resonant Waveguide Gratings (RWG)	Capable of investigating biological samples, nanoparticles and self-assembled nanostructured coatings; High throughput	Needs a skilled operator; Operator variable; Expensive equipment	[232,233,234]

## Data Availability

No new data were created or analyzed in this study. Data sharing is not applicable to this article.
